# Production and characterization of homologous protoporphyrinogen IX oxidase (PPO) proteins: Evidence that small N-terminal amino acid changes do not impact protein function

**DOI:** 10.1371/journal.pone.0311049

**Published:** 2024-09-26

**Authors:** Cunxi Wang, Meiying Zheng, Chandler Est, Remi Lawal, Wenguang Liang, David A. Korasick, Michael J. Rau, Scott A. Saracco, Virginia Johnson, Yanfei Wang, Tommi White, Wenze Li, Jun Zhang, Xin Gu, Flora Liu-Gontarek

**Affiliations:** 1 Regulatory Science, Bayer Crop Science, Chesterfield, Missouri, United States of America; 2 Plant Biotechnology, Bayer Crop Science, Chesterfield, Missouri, United States of America; 3 Small Molecules, Bayer Crop Science, Chesterfield, Missouri, United States of America; Uppsala University, SWEDEN

## Abstract

Transgenic soybean, cotton, and maize tolerant to protoporphyrinogen IX oxidase (PPO)-inhibiting herbicides have been developed by introduction of a bacterial-derived PPO targeted into the chloroplast. PPO is a membrane-associated protein with an intrinsic tendency for aggregation, making expression, purification, and formulation at high concentrations difficult. In this study, transgenic PPO expressed in three crops was demonstrated to exhibit up to a 13 amino acid sequence difference in the N-terminus due to differential processing of the chloroplast transit peptide (CTP). Five PPO protein variants were produced in and purified from *E*. *coli*, each displaying equivalent immunoreactivity and functional activity, with values ranging from 193 to 266 nmol min^-1^ mg^-1^. Inclusion of an N-terminal 6xHis-tag or differential processing of the CTP peptide does not impact PPO functional activity. Additionally, structural modeling by Alphafold, ESMfold, and Openfold indicates that these short N-terminal extensions are disordered and predicted to not interfere with the mature PPO structure. These results support the view that safety studies on PPO from various crops can be performed from a single representative variant. Herein, we report a novel and robust method for large-scale production of PPO, enabling rapid production of more than 200 g of highly active PPO protein at 99% purity and low endotoxin contamination. We also present a formulation that allows for concentration of active PPO to > 75 mg/mL in a buffer suitable for mammalian toxicity studies.

## Introduction

Yield loss due to weed pressure is one of the greatest limiting factors to efficient crop production [1]. The development of herbicide-tolerant crops has led to striking advancements in weed management, including an estimated $21.7 billion in additional farm income and a 225,000 metric ton reduction in herbicide active ingredient use from 1996 to 2012 [2]. Accordingly, genetically modified (GM) crops tolerant to herbicides have been rapidly adopted by farmers worldwide. From 2013 to 2020, farm income gains have continued to grow, tripling compared to the period from 1996 to 2012 [3]. This shift has also resulted in a significant reduction in the release of greenhouse gas emissions from tillage and herbicide use [4]. While GM crops tolerant to herbicides such as glyphosate and glufosinate have proven valuable in a commercial setting, crops with new herbicide-resistant traits are needed to avoid overreliance on any single herbicidal mode of action and to increase available options for managing difficult to control weed species.

Protoporphyrinogen IX oxidase (PPO) catalyzes the oxidation of protoporphyrinogen IX to protoporphyrin IX, the last shared reaction in the biosynthesis pathways of heme and chlorophyll [5]. In terrestrial plants, the PPO enzyme is the target of a large and diverse family of PPO-inhibiting herbicides for weed management [6–9]. Upon exposure to PPO-inhibiting herbicides, plants experience a build-up of the reactive protoporphyrinogen IX intermediate that escapes from the chloroplast into the cytoplasm where it is non-specifically oxidized to protoporphyrin IX. Interactions with O_2_ and light result in formation of singlet O_2_ radicals which cause peroxidative damage to membrane lipids and subsequent plant cell necrosis.

Three classes of nonhomologous, isofunctional PPO enzymes have been identified: HemY, HemJ, and HemG [10–12]. HemY, the PPO present in terrestrial plants and the target of PPO-inhibiting herbicides, is ubiquitous in both prokaryotes and eukaryotes. HemY PPO is an oxygen dependent, membrane bound dimer that utilizes flavin adenine dinucleotide (FAD) as a cofactor for its activity [13, 14]. HemJ is thought to originate in α-proteobacteria but is also present in other proteobacteria and cyanobacteria. HemJ PPO is a membrane-bound oligomer and does not require oxygen or menadione as an electron acceptor, differentiating it from HemY or HemG [15, 16]. HemG occurs primarily in γ-proteobacteria. HemG PPO is an oxygen-independent enzyme that forms membrane-associated oligomers and uses non-covalently bound flavin mononucleotide (FMN) as a co-factor [10, 11].

It has been demonstrated that expression of a bacterial HemG-type PPO derived from *Enterobacter cloacae* (H_N90 PPO, hereafter PPO) in maize, cotton, and soybean chloroplasts confers tolerance to applications of PPO-inhibiting herbicides [7]. In these transgenic plants, PPO is targeted to the chloroplast with a plant-derived chloroplast transit peptide (CTP) at the N-terminus, which protects against the effects of transgenic plant exposure to PPO-inhibiting herbicides [7, 8]. Due to differential processing of the CTP preceding PPO in these plant species (maize, cotton, and soybean), transgenic PPO exhibits small differences (up to 13 residues) in N-terminal amino acid sequence. Incomplete cleavage of the CTP is frequently observed in transgenic proteins targeted to the chloroplast, but to date these differences have shown no observable impact on transgenic protein function or safety [17, 18]. We hypothesize that these small differences in amino acid sequence at the N-termini do not impact PPO functional properties, as these PPO variants all display a PPO-inhibiting herbicide resistance phenotype *in planta* [7].

A thorough safety assessment of transgenic PPO expressed in GM crops for weed management must be conducted prior to commercialization. Guidance for assessing the safety of newly expressed proteins in a GM crop (trait proteins) has been developed and standardized over the past 30 years [19–22]. To align with these guidelines, large quantities (up to 200 grams (g)) of each trait protein are needed for various safety studies. However, the expression level of these trait proteins in transgenic crops is very low (often less than 1 ppm (part per million) in grain tissue). To produce 200 g of purified protein would require more than 200,000 kg of plant material, assuming a 100% yield recovery. Therefore, safety studies are conducted with equivalent trait proteins produced using heterologous expression systems such as *E*. *coli*.

Although heterologous expression of trait proteins can alleviate the issue of low expression *in planta*, intrinsic protein characteristics can still present challenges for purification. PPO is a membrane-associated protein and has a propensity for aggregation that limits formulation at high concentration [11, 23, 24]. Preliminary screening of *E*. *coli*-derived transgenic PPO suggests a similar tendency for aggregation, making production of ~200 g of PPO protein at a suitable purity and concentration level for safety studies a challenging task. Herein, we present a scalable method for purification of functionally active PPO by use of an N-terminal 6xHis-tag, and we apply this method to purify 200 grams of *E*. *coli*-derived PPO for use in safety assessments.

We hypothesize that these homologous PPO variants expressed across different transgenic crops have equivalent physicochemical and functional properties to the ‘wild-type’ (bacterial *E*. *cloacae* H_N90) or mature PPO as well as the corresponding sequence-matched His-tagged version. If this hypothesis is valid, then we suggest one safety study package conducted using mature PPO is applicable for all PPO N-terminal variants, precluding the need for a complete *de novo* safety assessment package for each variant.

## Materials and methods

Unless otherwise stated, all reagents were purchased from Sigma-Aldrich (Burlington, MA, United States of America) and all chromatography equipment, resins and prepacked columns were purchased from Cytiva (Marlborough, MA, United States of America).

### Development of transgenic crops tolerant to PPO-inhibiting herbicides

**Fig 1:**
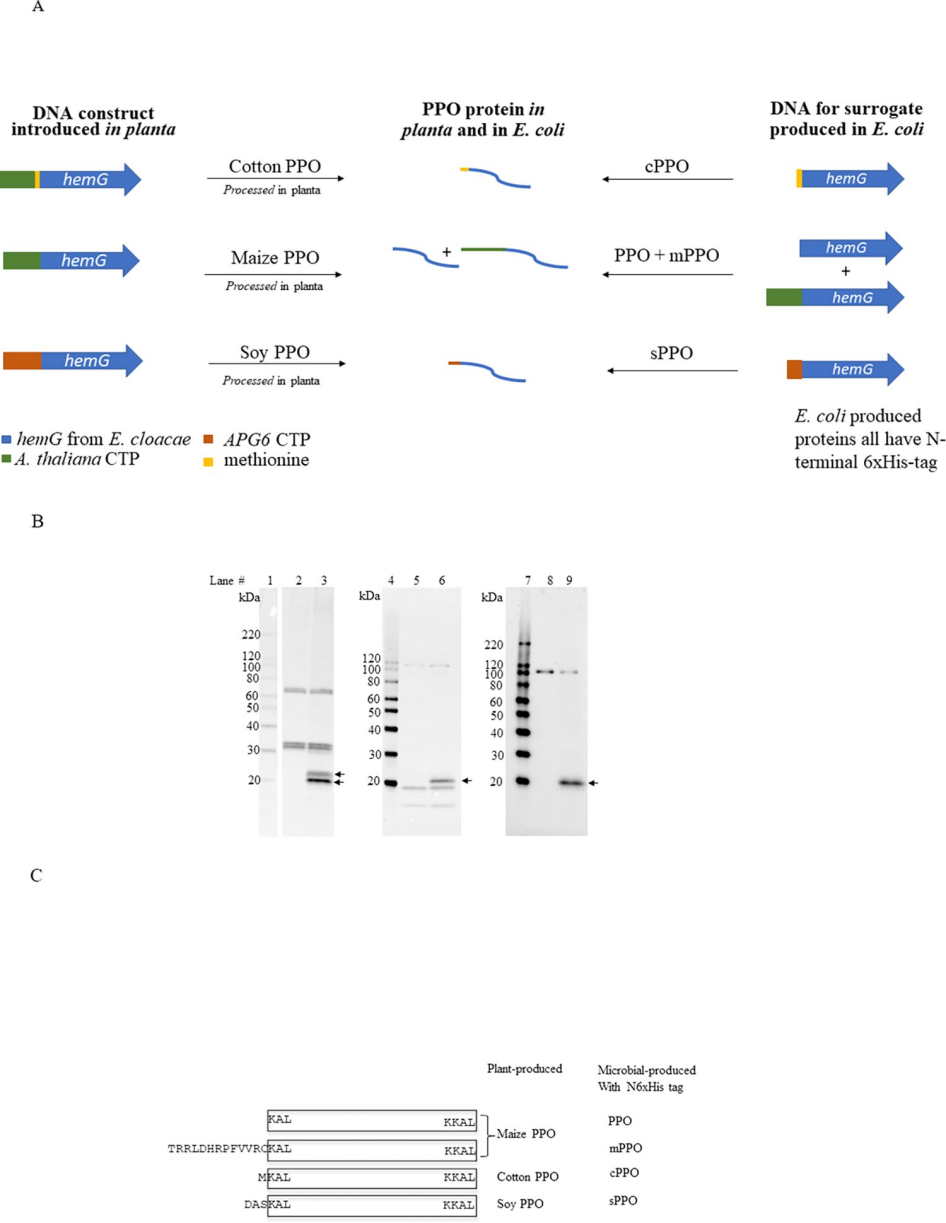
**A: Schematic of various PPO constructs produced and tested. B: Western blot analysis of PPO protein extracted from plant sources.** Lanes 1, 4 & 7: MagicMark XP Western Protein Standards (Thermo Fisher Scientific); Lanes 2 & 3: wild-type and PPO-transgenic maize seeds, respectively; Lanes 5 & 6: wild-type and PPO-transgenic cotton seeds, respectively; Lanes 8 & 9: wild-type and PPO-transgenic soybean leaf tissues, respectively. Arrows on blot indicate specific PPO proteins (S1 Raw images). **C: N-terminal sequence of plant produced PPO**.

Transgenic maize, cotton, and soybean plants tolerant to PPO-inhibiting herbicides were developed by expressing H_N90 PPO (*hemG*) from *E*. *cloacae* (GenBank accession number MN102108) with CTPs appended to the N-termini to direct the protein into the chloroplast. In maize, PPO was fused with a CTP originating from the Albino and pale green 6 (*APG6*) gene from *Arabidopsis thaliana*; cotton PPO was designed similarly, but a methionine was retained between the CTP and the PPO coding sequence [7, 25]. Transgenic soybean was created by expressing the same PPO sequence but fused with the N-terminal *APG6* CTP (WWB02972.1, partially synthetic peptide). Fig 1 illustrates the various forms of PPO present in the transgenic crops, in which PPO in maize, cotton and soybean are designated as maize PPO, cotton PPO and soy PPO, respectively. The microbially produced PPO variants with N-terminal 6xHis-tags are named PPO, mPPO, cPPO and sPPO, respectively. H_N90 PPO (the gene product of *hemG* in *E*. *cloacae*) expressed without a 6xHis-tag is designated as tag-free PPO.

### Isolation of plant-derived PPO for N-terminal sequence determination

#### Cotton PPO

Cotton PPO was extracted from 10 g of ground and defatted cotton seed powder by 50 mM Tris, pH 7.4, 10 mM (NH_4_)_2_SO_4_, 5 mM Benzamidine, 1 mM EDTA, 1 mM PMSF, 2 mM DTT, 10% glycerol, 0.5% lauryl maltose neopentyl glycol (LMNG), 0.075% Soy-L-α-phosphatidylcholine (PC), 0.025% cholesteryl hemisuccinate (CHS) and protease inhibitors at a ratio of 1 g powder/20 mL buffer. The extracted protein was loaded onto a Capto Q column and eluted using a step salt gradient of 0.3 M NaCl. Ammonium sulfate was added to the Q fraction to a final concentration of 0.9 M and then loaded onto a Butyl Sepharose column. The protein was eluted with a buffer of 50 mM Tris, pH 8, 10% glycerol, 2 mM Benzamidine, 0.25% LMNG, 0.0375% Soy-PC and 0.0125% CHS. The eluent containing PPO was further purified by immunoaffinity chromatography (IAC) using a PPO monoclonal antibody (mAb, Silver Lake Research, Irwindale, CA), crosslinked to Protein A resin using bis(sulfosuccinimidyl) suberate (Thermo Fisher Scientific, Waltham, MA).

#### Soy PPO

Soy PPO was extracted from 350 g of hexane-defatted soybean seed flour by 50 mM Tris, pH 8.0, 5 mM CHAPS (3-((3-cholamidopropyl) dimethylammonio)-1-propanesulfonate), 1 mM EDTA, 2 mM DTT, 4 mM benzamidine-HCl, 10% glycerol and protease inhibitors at a ratio of 1 g powder/30 mL buffer. The extracted protein was loaded onto a DEAE-Sepharose Fast Flow column and eluted using a linear salt gradient from 0 to 0.5 M NaCl. The eluents were concentrated, buffer exchanged and further purified by IAC as described above.

#### Maize PPO

The isolation of maize PPO utilized the method described for cotton PPO. PPO was isolated from ~150 g of maize grain.

These PPO proteins isolated from plant tissues were subjected to N-terminal sequence analysis using the method described below.

### Small scale (mg quantity) and large-scale (g quantity) His-tagged PPO protein production from *E*. *coli*

The coding sequences for PPO from maize, cotton, and soybean were cloned into pET24 vectors (Novagen, Madison, WI) with a non-cleavable, N-terminal 6xHis-tag. After sequence verification, these PPO plasmids were transformed into BL21(DE3) *E*. *coli* (New England Biolabs, Ipswich, MA) and expressed in auto-induction media (AIM) supplemented with 50 μg/mL kanamycin at 37°C for 4 hours, followed by overnight induction at 16°C. The cell culture was harvested the next day, and the cell paste was stored at -80°C for purification.

Fifteen conditions were screened to improve purification and recovery for the His-tag PPO, encompassing various buffers, salt concentrations, imidazole concentrations, FMN, and detergent concentrations, including three types of detergents (CHAPS, Triton X-100, and Thesit) for chromatography. Additionally, 10 conditions were screened for long-term storage, involving arginine (Arg), histidine (His), and imidazole. Furthermore, 50 conditions were screened to enable concentration of PPO to higher than 75 mg/mL, covering different pH levels and buffer additives, such as salts, arginine, glutamate, histidine, glycerol, detergents, and FMN.

After optimizing the purification method (the optimized purification method was summarized in S3 Table), all His-tagged PPO variants, including PPO, cPPO, mPPO, and sPPO, were produced using the same cell lysis buffer, chromatography resin, and conditions for loading, washing, and elution. The purification method proved to be robust and easily scalable. For the purification of mg (small) quantities of cPPO and mPPO needed for protein characterization, cells were lysed by sonication for 5 minutes in an ice bath, while a microfluidizer was used to lyse *E*. *coli* cells for large-scale production of PPO. Chromatography for small-scale purification was carried out using a HiScale 16/20 column equipped on an AKTA Pure system, while the large-scale production utilized a BPG 300/500 column equipped on an AKTA Pilot system.

The large-scale His-tagged PPO purification process is as follows: *E*. *coli* cell paste (~2.5 kg) was re-suspended in IMAC (immobilized metal-affinity chromatography) Extraction Buffer, consisting of 50 mM HEPES, 300 mM NaCl, 2 mM MgCl_2_, 10 mM imidazole, 0.1 mM FMN, 10% (v/v) glycerol, 2% (w/v) Thesit, Benzonase (100 U/mL), Lysozyme (100 μg/mL), 2 mM Benzamidine, 1 Tablet of Complete Protease inhibitor per 100 mL, at pH 7.5, at a ratio of 20 L of IMAC Extraction Buffer per kg of cell paste. Cell lysis was performed at approximately 20,000 PSI (pounds per square inch) using an M-110EH microfluidizer (Microfluidics, Westwood, MA). All subsequent PPO handling was conducted at 4°C. Following lysis, the crude lysate was centrifuged at 15,000 x g for 30 minutes, and the supernatant was collected for IMAC.

The His-tagged PPO was bound to 8 L of Ni-NTA resin (Cube Biotech, Monheim, Germany) for 1.5 hours via a batch process under gentle stirring, after which the resin was collected by filtration with Miracloth. The PPO-bound resin was divided and loaded onto two BPG 300/500 columns and packed using IMAC Wash Buffer 1 (50 mM HEPES, 50 mM NaCl, 15 mM imidazole, 0.1 mM FMN, 20% (v/v) glycerol, 1% (w/v) Thesit, pH 8.0) by an AKTA Pilot. All subsequent purification steps were performed using the AKTA Pilot. The PPO-bound resin was washed with 10 column volumes (CV) of IMAC Wash Buffer 1 at a flow rate corresponding to a residence time of 11.8 minutes. A second wash was performed with 5 CV of IMAC Wash Buffer 2 (50 mM HEPES, 50 mM NaCl, 20 mM imidazole, 0.1 mM FMN, 20% (v/v) glycerol, 0.1% (w/v) Thesit, pH 8.0) at a flow rate corresponding to a residence time of 11.8 minutes. PPO was eluted with 5 CV of IMAC Elution Buffer (11 mM phosphate buffer (pH 7.4 to 7.6), 135 mM NaCl, 2.7 mM KCl, 56 mM L-arginine, 80 mM L-histidine, 60 mM imidazole, 0.1 mM FMN, 20% (v/v) glycerol, 0.1% (w/v) Thesit, pH 9.0) at a flow rate corresponding to a residence time of 14.1 minutes. The eluate was collected from both columns, and fractions containing PPO were identified by the chromatogram signal at 280 nm and pooled. PPO samples were aliquoted on dry ice and placed directly at -80°C for long-term storage.

For PPO concentration or formulation, the purified PPO was diafiltrated against a buffer containing 5 mM monosodium phosphate, 28 mM L-arginine, 0.1 mM FMN, at pH 10.3 for 10 turnovers and then concentrated to the expected concentration as determined by Bio-Rad Bradford protein assay (Bio-Rad, Hercules, CA). This process was conducted using an ultrafiltration hollow fiber cartridge (100 kDa molecular weight cutoff, 1.15 m^2^, Cytiva).

### Tag-free PPO purification from *E*. *coli* (μg quantity)

For small-scale tag-free PPO purification from *E*. *coli*, the coding sequence corresponding to cotton PPO was cloned into a pET24 vector (Novagen, Madison, WI) and transformed into BL21(DE3) *E*. *coli* (New England Biolabs). Tag-free PPO was expressed using the same conditions as that for His-tag PPO expression. *E*. *coli* cells expressing tag-free PPO were pelleted, pooled and re-suspended in IAC Extraction Buffer (50 mM Tris, 300 mM NaCl, 0.1 mM EDTA, 0.1 mM FMN, 0.1% (*w/v*) Thesit, pH 8.0) supplemented with Benzonase (75 U / mL) and Lysozyme (100 μg / mL) at a ratio of 5 mL of IAC Extraction Buffer/g of cell paste. Cells were lysed by sonication for 5 minutes in an ice bath, then diluted 2-fold with the IAC Extraction Buffer. All subsequent PPO handling was performed at 4°C. Tag-free PPO was extracted from the lysed cells for 30 minutes with gentle agitation. Following extraction, the crude lysate was centrifuged at 5,000 *x g* for 15 minutes, and the supernatant collected for IAC.

Tag-free PPO was purified in one step using the mAb affinity method described for plant PPO purification. Binding occurred for 2 hours via batch process, after which the resin was collected by centrifugation at 1,000 *x g* for 3 minutes. The PPO-bound resin was loaded into a HiScale 16/20 column and packed using IAC Extraction Buffer by an AKTA Pure. The PPO-bound resin was washed with 10 CV of IAC Wash Buffer (50 mM Tris, 825 mM NaCl, 0.1 mM EDTA, 0.1 mM FMN, 0.1% (*w/v*) Thesit, 12.5% (*v/v*) propylene glycol, pH 8.0) at a flowrate corresponding to a residence time of 2.3 minutes. Tag-free PPO was eluted with 5 CV of IAC Polyol Elution Buffer (50 mM Tris, 1 M NaCl, 0.1 mM EDTA, 0.1 mM FMN, 0.1% (*w/v*) Thesit, 50% (*v/v*) propylene glycol, pH 8.0) at a flowrate corresponding to a residence time of 8 minutes. The eluate was collected in 0.5 mL fractions in a 96-well Masterblock, with each well pre-loaded with 1.5 mL of Dilution Buffer (50 mM Tris, 100 mM NaCl, 0.033 mM EDTA, 0.033 mM FMN, 0.033% (*w/v*) Thesit, pH 8.0) to limit the duration of PPO exposure to IAC Polyol Elution Buffer. Fractions containing tag-free PPO were identified by the chromatogram signal at 280 nm, pooled and immediately concentrated & buffer exchanged into IAC PPO Storage Buffer (11 mM phosphate, 135 mM NaCl, 2.7 mM KCl, 56 mM L-arginine, 0.1 mM FMN, 80 mM L-Histidine, 60 mM imidazole, 0.1% (*w/v*) Thesit, pH 9.0) by centrifugation at 4,000 *x g* in a 10 kDa Amicon MWCO filter (Millipore). Once tag-free PPO had been concentrated, the protein was diluted 2-fold with IAC PPO Storage Buffer supplemented with 40% (*v/v*) glycerol, for a final total glycerol concentration of 20% (*v/v*) as previously described for His-tagged PPO. Tag-free PPO samples were aliquoted and placed directly at -80°C for long-term storage.

### Characterization of PPO

All methods utilized to characterize PPO protein were previously reported [18, 26, 27] with some minor changes. Briefly, the mature PPO concentration was determined by amino acid compositional analysis (AAA). For AAA, Amino Acid Standard H (Thermo Fisher Scientific, Waltham, MA) was used for generation of a standard curve, and α-aminobutyric acid (Sigma-Aldrich) was used as an internal calibrant. Protein was hydrolyzed with 6 N HCl, derivatized with AccQ-Fluor Ultra reagent (Waters Corporation, Milford, MA) and analyzed using an Acquity UPLC Module (Waters Corporation) equipped with a reverse-phase AccQ-Tag™ column (Waters Corporation). The molecular weight and purity-corrected protein concentrations for the *E*. *coli-*produced other PPO variants were determined using gel-based densitometry analysis. Variants were resolved on a pre-cast Tris-Glycine 4–20% (*w/v*) polyacrylamide gradient mini-gels by Tris-Glycine-SDS running buffer (Invitrogen, Carlsbad, CA) and stained by Colloidal Brilliant Blue G. For western blot analysis of *E*. *coli* produced PPO proteins, each purified protein sample was resolved on a pre-cast Tris-Glycine 4–20% (*w/v*) polyacrylamide gradient mini-gel by Tris-Glycine-SDS running buffer (Invitrogen, Carlsbad, CA) and electro-transferred onto a nitrocellulose membrane using the Bio-Rad Transblot-Turbo system (Bio-Rad). After washing, the membrane was blocked and processed on an iBind Card in an iBind Western Device (Thermo Fisher Scientific) according to the manufacturer’s protocol. The blot was probed with a mouse anti-PPO mAb (Silver Lake Research, Irwindale, CA) and imaged with a secondary antibody conjugated to horseradish peroxidase (Vector Labs, Newark, CA). Imaging was performed with a Chemidoc Imager (Bio-Rad) using SuperSignal ECL substrate (Thermo Fisher Scientific). To determine the PPO expression profile in GM crops, total protein from transgenic seed (maize and cotton) or leaf (soybean) was extracted directly using Laemmli buffer and then subjected to western blot analysis similar to *E*. *coli* produced PPO proteins.

Samples for the PPO activity assay were prepared on ice, but the assay was performed at room temperature using a modified protocol based on Larue et al 2020. Briefly, PPO variants were diluted to 0.025 mg/mL in Dilution Buffer (100 mM Tris, 1 mM EDTA, 200 μM FMN, 0.1% (*w/v*) Tween 80, pH 7.4). Protoporphyrin (PPN) IX standard was diluted to 2.5 μM using Standard Dilution Buffer (100 mM Tris, 1 mM EDTA, 2 mM reduced glutathione, 50 μM menadione, 8 μM FMN, 0.1% (*w/v*) Tween 80, pH 7.4). A standard curve was generated using a 6-step standard dilution of PPN IX ranging from 0.078–2.5 μM prepared by serial two-fold dilution. 0.2 μg of PPO protein in 8 μL of sample Dilution Buffer was mixed with 190 μL of reaction solution (100 mM Tris, 1 mM EDTA, 2 mM reduced glutathione, 50 μM menadione, 0.1% (*w/v*) Tween 80, pH 7.4) for each reaction. The reaction was initiated by adding 2 μL of 240 μM protoporphyrinogen (PPG) IX, and fluorescence was measured with a SpectraMax M2 Microplate Reader (Molecular Devices, Sunnydale, CA, USA) every 30 seconds for a total of 6 minutes using an excitation wavelength of 405 nm and an emission wavelength of 630 nm. The PPO enzyme specific activity is reported as the average conversion rate of PPG IX to PPN IX during the 6 minutes (nmol min^-1^ mg^-1^ of PPO protein).

The Charles River Endosafe® nexgen-MCS™ system (Charles River Laboratories, Cleveland, USA) was utilized to measure endotoxin levels in the PPO protein sample. This advanced system employs the Limulus Amebocyte Lysate (LAL) assay with kinetic chromogenic detection, enabling rapid and accurate endotoxin quantification. The sample was diluted with endotoxin-free water, and 25 μl of the diluted sample was carefully loaded into Endosafe® cartridges containing pre-loaded reagents. These loaded cartridges were then inserted into the nexgen-MCS™ unit, initiating the automated assay process. Results were obtained within 15–20 minutes, displaying precise endotoxin concentrations in EU/mL, which were subsequently calculated to EU/mg of protein (one EU (endotoxin unit) is approximately equivalent 0.1 to 0.2 ng of *E*. *coli* lipopolysaccharide (LPS)). Prior to measurement, the calibration of the nexgen-MCS™ system was performed to ensure its performance and accuracy.

### PPO identity confirmation

The identities of all PPO variants including plant and *E*. *coli*-produced were confirmed by N-terminal sequence determination and peptide mass fingerprint analysis using nano liquid chromatography tandem mass spectrometry (LC-MS/MS). LC-MS/MS analysis was performed using a Dionex 3000 Ultimate nano-LC system (Thermo Fisher Scientific) interfaced to an Orbitrap fusion mass spectrometer (Thermo Fisher Scientific) which is equipped with a nano-ESI (electrospray ionization) source. For LC-MS/MS analysis, aliquots of the protein samples were separated by SDS-PAGE and the bands corresponding to PPO were excised from the gel, destained, reduced, alkylated and then digested with proteases i.e. trypsin, chymotrypsin and/or Asp-N (Promega Corporation, Madison, WI). The peptide digests were extracted, dried down and re-dissolved into 0.1% formic acid in water, and injected into the MS for analysis. Peptides were separated online using an Acclaim PepMap100 C18 nano column (75 μm id × 150 mm, 2 μm, 100 Å, Thermo Fisher Scientific). The LC-MS/MS dataset was searched against the theoretical PPO protein sequence. The mass tolerances of MS1 mass and MS/MS mass were set as 5 ppm and 0.5 Da, respectively and peptide charge was set at +2, +3, and +4. Carbamidomethylation of cysteine was set as a fixed modification and methionine oxidation was set as variable modification. The minimum length for peptides was set to five amino acids and a maximum of two missed cleavages for the enzyme was allowed.

N-terminal amino acid sequences for plant produced PPO were also determined by Edman degradation when the amount and purity of PPO proteins produced met Edman method requirements. For Edman degradation, an aliquot of each protein sample was subjected to SDS-PAGE analysis and then transferred to a PVDF membrane by electroblotting. The proteins transferred to the PVDF membrane were visualized by staining with Coomassie Brilliant Blue R-250, and the band containing full-length PPO protein was analyzed by Edman degradation. The analysis was performed for 10 to 15 cycles using automated Edman degradation chemistry [28]. A Shimadzu protein sequencer PPSQ-53A system (Shimadzu, Kyoto, Japan) was used with gradient elution for analysis of the PTH-amino acids. LabSolutions PPSQ software was used for data acquisition and processing. Chromatographic separation was performed on a Wakopak Wakosil PTH-GR (S-PSQ) (250 mm × 2.0 mm I.D.) column at 35°C. PTH-AA Standard Solution (FUJIFILM Wako) was used to chromatographically calibrate the instrument for analysis. A control protein (20 picomole β-lactoglobulin, Sigma-Aldrich) was analyzed before the analysis of the PPO protein. When protein purity and/or quantity purified from grain did not meet requirement for Edman degradation, N-terminal sequence of the plant-produced PPO was analyzed using LC-MS/MS as described above. In addition to enzyme digestion described above, the plant PPO samples were also digested chemically with 3 N hydrochloric acid (Thermo Fisher Scientific) for LC-MS/MS analysis.

### PPO structure modeling

Numerous conditions to crystallize and perform structure determination using X-ray Diffraction of *E*. *coli* expressed PPO were attempted using multiple vapor diffusion methods and under oil. Although yellow crystals were obtained, X-ray diffraction was not observed, and experimental structure determination was not successful. Therefore, three *in silico* prediction programs, Alphafold2 [29], ESMfold [30] and Openfold [31], were used to compare and model the various PPO variants. Structures were analyzed in PyMOL v2.5.8 (https://pymol.org/). RMSD (root mean square deviation) value ranges were determined by comparing output pdb files of these *in silico* prediction tools, where the Alphafold model (ranked0.pdb) served as the reference when using the PyMOL “Alignment” plugin. S1 Table displays five PPO variant sequences submitted for protein modeling.

## Results

### PPO expression and identity in transgenic crops

Maize, cotton, and soybean plants expressing transgenic PPO targeted to chloroplasts each demonstrate tolerance to PPO-inhibiting herbicides [7]. Western blotting of plant extracts shows that transgenic maize accumulates two PPO variants with apparent molecular weights of ~19 and ~21 kDa (Fig 1B, lane 3). Additional bands migrating at ~32, 34 and ~70, 72 kDa were also detected; however, these bands are detected in wild-type tissue as well, indicative of non-specific antibody binding (Fig 1B, lane 2). To further characterize these two maize PPO variants, both proteins were partially purified from grain by serial chromatography via ion exchange, hydrophobic interaction, and immunoaffinity enrichment. Purified PPO was resolved by SDS-PAGE and characterized by Edman sequencing and mass spectrometry analysis. The data confirm that the ~19 kDa variant corresponds to the mature PPO protein (Fig 1C and S1 File), whereas the ~21 kDa variant consists of mature PPO containing 13 additional N-terminal amino acids (TRRLDHRPFVVRC) originating from the *Arabidopsis thaliana* APG6 CTP (Fig 1C and S1 File).

In contrast to transgenic maize, only one form of PPO was detected by western analysis in transgenic cotton grain extract, with an apparent molecular weight of ~20 kDa (Fig 1B, lane 6). Like the isolated protein sample from maize, western blotting also detected several non-specific bands (Fig 1B, lane 5) of both low (~12, ~18 kDa) and high molecular weights (~90, ~110 kDa) present in both transgenic and non-transgenic samples. Edman sequencing revealed the CTP was completely processed from the cotton PPO, leaving an N-terminal methionine (M) that was introduced for improved cotton PPO expression (Fig 1C and S1 File).

Similar to transgenic cotton, western blotting suggests only one form of PPO accumulates in transgenic soybean leaf tissues (the PPO expression level in soybean seed is too low to be clearly detected by western analysis without further enrichment), with a molecular weight of ~20 kDa (Fig 1B, lane 9). An additional, nonspecific band migrating at ~100 kDa was also detected (Fig 1B, lane 8). Mass spectrometry analysis confirms transgenic soy PPO contains three extra N-terminal amino acids (DAS) originating from the partially synthetic *APG6* CTP (Fig 1C and S1 File).

The H_N90 PPO protein shares less than 18% amino acid sequence identity with the endogenous PPO proteins from soybean and maize annotated in GenBank (GenBank #: BAA76348, AAG00946.1 and AAF26417.1). Consequently, the mAb raised against the H_N90 PPO protein did not recognize the endogenous PPO proteins in these plants. This is demonstrated in Fig 1B, where no band at the expected size for plant endogenous PPO (55 to 60 kDa) was observed.

### Expression screening and purification of active PPO proteins

Because safety assessment studies require large quantities of PPO, but the expression levels *in planta* are low (< 1 ppm). *E*. *coli* was explored for suitability as a host for heterologous expression of PPO. To ensure sequence identity between PPO derived from *E*. *coli* and PPO detected *in planta*, the amino acid sequence corresponding to transgenic PPO detected *in planta* was cloned into a variety of expression vectors utilizing tag-free or removable purification tags. These tags included His, SUMO (Thermo Fisher Scientific), Mistic (Addgene, Watertown, MA), Thioredoxin (Addgene) and CL7/Im7 (TriAltus Bioscience, Birmingham, MA) tags with respective protease cleavage sites to screen for the feasibility of tag-free PPO production. Since it is well established that the expression of soluble, active protein can be cell line and culture dependent, several *E*. *coli* cell lines, growth media, and temperatures were evaluated for efficiency in enhancing expression of soluble PPO. Additionally, detergents (up to 8 detergents tested for removable tag constructs) and other additives (glycerol, amino acids, salts, reducing agents and varying pH) were tested to improve solubility of *E*. *coli*-produced tagged PPO and subsequent efficiency for removal of tags (S2 Table). Overall, more than 700 different conditions were tested to produce tag-free PPO.

For the tag-free construct, soluble expression of PPO in all *E*. *coli* cell lines and growth conditions was low, and traditional chromatographic techniques including ion exchange or hydrophobic interaction, failed to purify the tag-free PPO.

For removable tagged constructs, SUMO- and Mistic-tagged PPO showed modest to high levels of expression, but the fused PPO accumulated in inclusion bodies and was not soluble with common buffers. Solubility of these fused PPO proteins was improved by incorporation of detergents and other additives (S2 Table). For His-, SUMO-, Thioredoxin- and CL7/Im7 tagged PPO, solubilization with 2% Thesit / 20% glycerol results in almost complete recovery of PPO. However, these solubilization conditions were ineffective for Mistic-tagged PPO, which only solubilized in the presence of 6 M urea. None of these tags facilitated enrichment of tagged PPO, and removal of the tags was highly inefficient (~1% for 6 M urea-solubilized Mistic-PPO, and ~ 250 mg of tag-free PPO from 250 L of culture for SUMO-tagged PPO). Thus, none of these conditions tested are feasible for large scale purification (up to 200 grams) of tag-free PPO needed for safety assessment studies.

We next evaluated an uncleavable 6xHis tag for large-scale PPO purification. For such constructs, both C- and N-terminal 6xHis tags were screened. Expression of PPO was high with an N-terminal His-tag, but markedly reduced with C-terminal His-tags (Table 1). The optimal expression system for N-terminally His-tagged PPO was found to be BL21(DE3) *E*. *coli* in an autoinduction medium at low temperature (18.6°C). Like the *E*. *coli-*endogenous PPO (accession# P0ACB4), which shares 59.8% amino acid identity to H_N90 PPO, the H_N90 PPO expressed from *E*.*coli* prefers a high concentration of salt (0.3 to 0.5 M NaCl) and detergent for its solubilization [11]. Among detergents tested during the purification process, including CHAPS, Triton X-100, and Thesit, Thesit performed the best for maintaining PPO activity. CHAPS reduced PPO activity during the isolation, and Triton X-100 failed to maintain PPO activity during freeze/thaw (Table 1).

**Table 1:** Summary of PPO production.

Activity	Key learnings	Comments
Constructs & Expression	The N-terminal 6xHis expressed efficiently, but the C-terminal 6xHis had low expression.	The expression level of the C-terminal 6xHis-tagged PPO is ~10% of that of the N-terminal 6xHis-tagged PPO.
Purification	PPO prefers high concentrations of NaCl (0.3 to 0.5 M) & detergent (2% Thesit) for initial solubilization, batch binding, column washing, and elution.	The combination of NaCl and Thesit resulted in solubilizing almost 100% PPO expressed from *E*. *coli*.
Endotoxin removal	High concentration of detergent (1% Thesit) was needed.	Thesit at <1% was less efficient in removal of endotoxin
Detergent optimization	Thesit > TritonX-100> CHAPS	PPO activity is approximately 70% in Triton X-100 and 30% in CHAPS in comparison to 100% activity in Thesit.
Activity & long-term storage	Arginine can replace NaCl for maintaining PPO activity in a storage buffer (1xPBS, 56 mM arginine, 0.1 mM FMN, 80 mM L-Histidine, 60 mM imidazole, 0.1% Thesit, 20% glycerol, pH 9.0)	The buffer components were optimized for eluting PPO from Ni-NTA resin and were effective for long term storage of PPO activity.
Buffer developed for formulation at >75 mg/mL	5 mM Sodium Phosphate, 28 mM Arg, 0.1 mM FMN, pH 10.3	The buffer components were demonstrated safe for animal studies while PPO activity is maintained at 100%
Yield	A total of 200 g of PPO protein was produced in three batches. Yield is ~2.5 g PPO/L cell culture and 8 g PPO/L resin	Purity of the PPO protein is 99%. Its activity (214 nmol min^-1^ mg^-1^) is equivalent to that from small scale production (Table 2). The endotoxin level was below 1 EU/mg of protein. Additionally, the protein remains stable when stored at -80°C for over six months.

**Fig 2:**
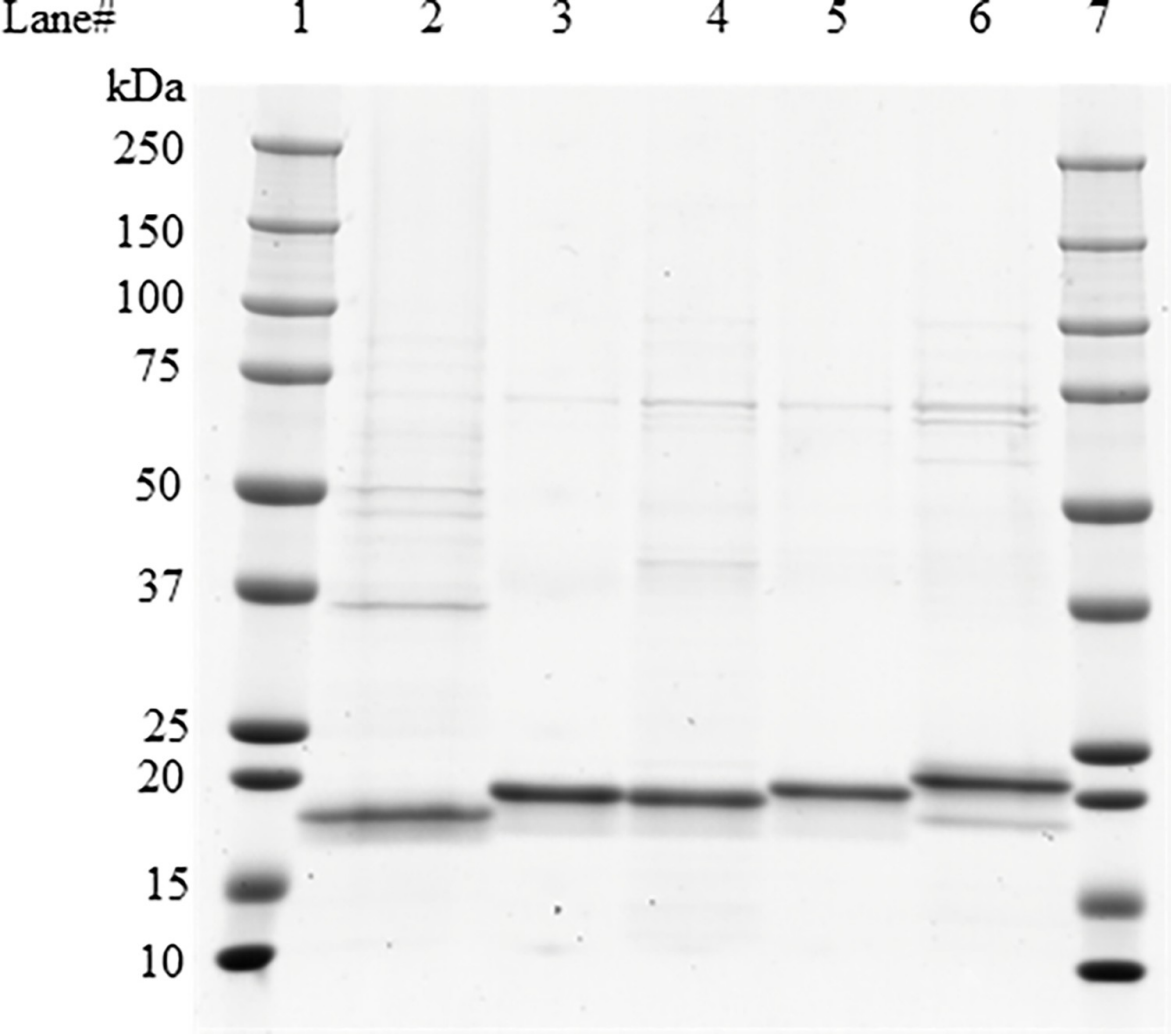
SDS PAGE analysis of PPO protein produced from *E*. *coli*. PPO proteins were resolved by SDS-PAGE. Each PPO variant was loaded in its respective lane with one μg of protein. Lanes 1 & 7: molecular weight markers (Precision Plus Protein Dual color, Bio-Rad), which were used to determine the apparent molecular weight of PPO variants; lane 2: Tag-free PPO; lane 3: PPO; lane 4: cPPO; lane 5: sPPO; lane 6: mPPO (S1 Raw images).

Imidazole is typically used for elution of protein from nickel affinity resin used for His-tagged protein purifications. However, high concentrations of imidazole were found to impact PPO activity (Table 1). Alternative elution conditions were screened to determine whether His-tagged PPO could be eluted at lower concentrations of imidazole. It was found that inclusion of L-histidine, L-arginine, and glycerol allowed for effective elution of PPO from the nickel resin at a lower concentration of imidazole and Thesit (Table 1). L-histidine is a common alternative to imidazole, as it competes with His-tagged proteins for binding sites on the nickel resin. However, elution buffers solely swapping imidazole for L-histidine show low recovery, due to precipitation of His-tagged PPO on-column. To overcome this issue, L-arginine and glycerol were included to enhance PPO solubility. L-arginine is known to improve membrane protein solubility and chromatographic recovery [32]. Using this strategy, ~25 mg of cPPO was purified from ~2.5 g of fermented *E*. *coli* cell pastes while ~7.3 mg mPPO was produced from ~ 2.4 g of fermented cell paste (Fig 2). To determine whether the N-terminal His-tag impacts PPO enzymatic activity, tag-free PPO was purified in low yields by a low-throughput, PPO specific mAb affinity method. However, due to the problems outlined above, only ~ 0.5 mg of the tag-free PPO was isolated from 10 g of cultured cells. Taken together, the expression and purification method described here represents significant improvement over traditional methods used for membrane-bound proteins.

### Characterization of PPO variants

Four PPO variants were purified from *E*. *coli* using an identical process utilizing Ni-NTA affinity resin while the tag-free PPO was isolated using the PPO specific mAb affinity. Data generated from the subsequent characterization of each protein are presented in Fig 2 and Table 2. Densitometric analysis of SDS–PAGE gels indicated these PPO were purified to >85% purity. Each PPO variant demonstrates the expected apparent molecular weight: tag-free PPO (18.4 kDa), PPO (19.6 kDa), mPPO (21.4 kDa), cPPO (19.6 kDa) and sPPO (20.2 kDa). The sequence identities of these purified PPO variants were confirmed by chymotryptic peptide mass fingerprinting using MS analysis, with >97% coverage of the entire protein sequence for all 5 forms (Table 2). The N-terminal and C-terminal sequences of all PPO variants were detected, all of which are consistent with the expected sequences of respective plant-produced PPO except addition of the N-terminal 6xHis tag.

**Table 2:** Summary of PPO characteristics.

Characteristics	Methods	Results				
		Tag-free PPO	PPO	mPPO	cPPO	sPPO
**Purity** ^**1**^	SDS PAGE/Densitometry	85%	99%	86%	93%	98%
**Apparent molecular weight (kDa)** ^**1**^	SDS PAGE/Densitometry	18.4	19.6	21.4	19.6	20.2
**N-terminal sequence** ^**2**^	Mass spectrometry	MKALVLYSTRDGQTH	MHHHHHHKALVLYSTR	MHHHHHHTRRL	MHHHHHHMKALVLYST	MHHHHHHDASKALVLY
**C-terminal sequence** ^**2**^	Mass spectrometry	KFAEDFAKLSYKKAL	KFAEDFAKLSYKKAL	KFAEDFAKLSYKKAL	KFAEDFAKLSYKKAL	KFAEDFAKLSYKKAL
**Mass fingerprint** ^**2**^	Mass spectrometry	100% coverage of expected sequence	100% coverage of expected sequence	97% coverage of expected sequence	100% coverage of expected sequence	100% coverage of expected sequence
**Activity**^**3**^ **(**nmol min^-1^ mg^-1^)	Spectrophotometer	193 ± 20	219 ± 37	266 ± 30	214 ± 14	200 ± 13

Supplementary data are available in supporting information files: S2 File^1^, S3 File^2^ and S5 Table^3^, respectively. ^3^Reported activity values represent the mean and standard deviation. The mean value was calculated from n ≥4.

**Fig 3:**
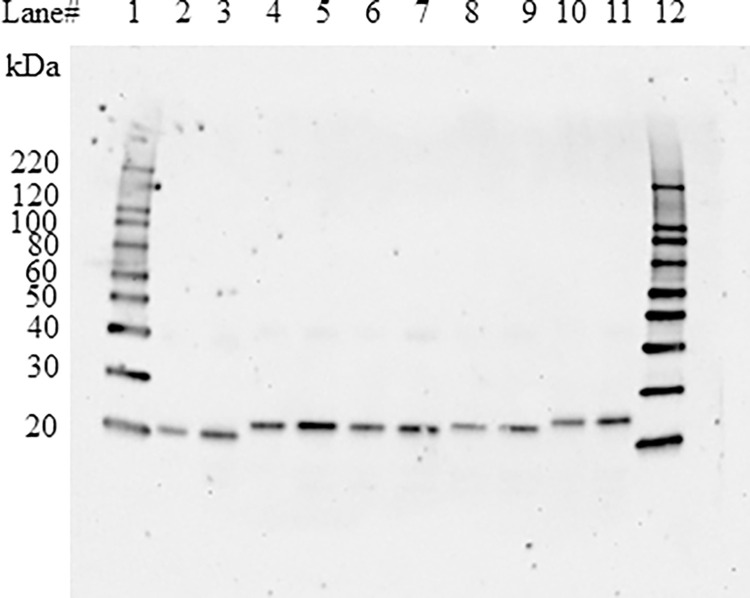
Western blot analysis of PPO protein produced from *E*. *coli*. PPO proteins were resolved on a pre-cast Tris-Glycine 4–20% (*w/v*) polyacrylamide gradient mini-gel by Tris-Glycine-SDS running buffer (Invitrogen, Carlsbad, CA) and electro-transferred onto a nitrocellulose membrane. The blot was probed with an anti-PPO specific mAb and developed using an enhanced chemiluminescence system. Either 0.75 or 1.5 ng of each PPO variant was loaded in each lane containing PPO protein. Lanes 1 and 12: MagicMark XP Western Protein Standards (Thermo Fisher Scientific); lanes 2 and 3: Tag-free PPO; lanes 4 and 5: PPO; lanes 6 and 7: cPPO; lanes 8 and 9: sPPO; lanes 10 and 11: mPPO (S1 Raw images).

Immunoblot analysis was conducted to determine the relative immunoreactivities of the PPO variants. The results demonstrate all PPO proteins migrate as expected (Fig 3) and show comparable band intensities, suggesting the PPO variants have equivalent immunoreactive properties that are not affected by the various small N-terminal extensions due to the His-tags and/or incomplete processing of the CTP sequences (Fig 3).

We next quantified the specific activity of each PPO variant, which ranged from 193 to 266 nmol min^-1^ mg^-1^ (Table 2). Although these PPO variants were isolated using different purification methods (mAb vs NTA affinity resin) and contain varying N-terminal sequences, they display similar functional activity. The observed variation in PPO activity falls within the assay variance (≤30%) and is well below the 2-fold criterion suggested elsewhere [23]. Our primary focus was on determining the PPO protein functional equivalence. Equivalence testing is particularly valuable for demonstrating the absence of a meaningful effect [33]. The prediction interval (PI) is a well-established statistical analysis method to set acceptance limits for functional equivalence testing [34–36]. The protein variants were considered to have equivalent functional activity if their specific activities fell within the acceptance limits of 117 to 280 nmol min^-1^ mg^-1^ as determined by the 95% PI calculated from a set of activity data generated using the mature PPO protein (S4 Table). Since the specific activities of these PPO variants fell within the PI, they were deemed to have equivalent functional activity. These data support the conclusion that small extensions of N-terminal amino acid sequence do not impact the functional activity of PPO. Additionally, the fact that the His-tag does not impact PPO functional activity is consistent with previously reported data [11, 23].

### Scale up of active PPO production with a single step chromatographic process for safety assessments

Protein safety assessments require hundreds of grams of active protein. Development of a scalable method is essential for large scale production of active PPO (Table 1). A method for large-scale purification of PPO was developed using an N-terminal 6xHis-tag as the N-terminal 6xHis does not impact PPO functional activity (S3 Table). To solubilize PPO from the heterologous *E*. *coli* host, relatively high salt (0.3 M NaCl) and detergent (2% Thesit) concentrations are required. Binding of solubilized 6xHis-tagged PPO to the Ni-NTA resin was performed in a batch mode process to facilitate rapid and well-distributed binding of PPO to the Ni-NTA resin bulk. A combination of L-arginine, L-histidine, and glycerol was used to elute PPO from the Ni-NTA resin. Initially, the large-scale purified PPO contained a high concentration of endotoxin, which can induce an immune response in mammals that interferes with animal toxicity studies. To remove the endotoxin, Ni-NTA-bound PPO was washed with 1% Thesit for 10 column volumes (CV) prior to elution of PPO. Using this optimized method (S3 Table), 200 g of 99% pure His-tagged PPO (Table 1) was purified from 80 L of *E*. *coli* fermentation paste. The large-scale purified PPO exhibits activity comparable to that from small-scale production (Table 1 and 2), with an endotoxin level below one EU/mg of protein.

High concentrations of protein solution (>75 mg/mL) are needed for animal toxicity studies, and PPO formulation at this concentration presents challenges due to the intrinsic propensity for PPO to aggregate. Surprisingly, PPO could be formulated to >75 mg/mL using a simple buffer consisting of only L-arginine and FMN in 5 mM sodium phosphate, components (Table 1) that should not affect mice in either extended dosing or acute toxicity studies at the levels used.

### Structural analysis

**Fig 4:**
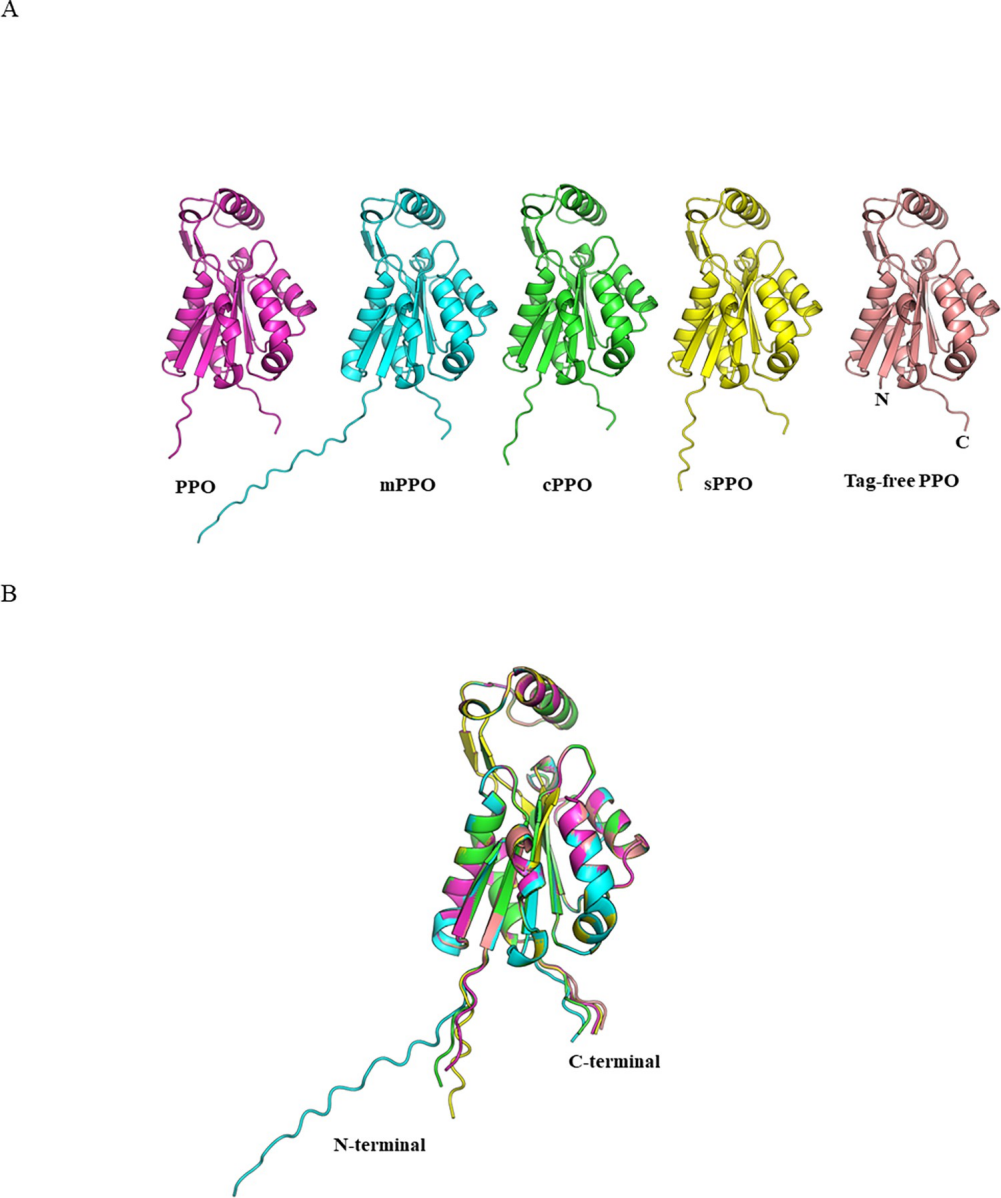
Structure alignment of PPO variants. The structures modeled using Alphafold are presented as representatives. A: Individual PPO variant structure; B: Structure alignment of PPO variants with the tag-free PPO. All align with RMSD of <0.3 Å.

The comparison of protein structures is a fundamental approach to understanding the biological, physical, and chemical properties of proteins and their various functionalities. Despite numerous attempts at determining the structure using both protein crystallography and CryoEM, an experimental structure could not be obtained. The structure of PPO variants and PPO was modeled using three *in silico* structure prediction software tools; Alphafold [29], ESMfold [30] and Openfold [31]. All five PPO protein structure models were in excellent alignment (Fig 4), as they only vary at their N-termini (S1 Table). Tag-free PPO lacks N-terminal extension or native start codon (Genbank: QHZ99190.1) and is used as the reference for comparison. RMSD values within each variant are reported using *in silico* predicted pdbs (five Alphafold ranked pdbs, one ESMfold pdb, and two Openfold pdbs) and Alphafold ranked_0.pdb was used as the reference. Tag-free PPO, PPO, cPPO, mPPO and sPPO predicted models comparing within each variant have an average RMSD of 0.25, 0.27, 0.25, 0.31, and 0.28 Å, respectively. Comparing RMSDs of the Alphafold ranked_0.pdb of each variant to the other, tag-free PPO versus PPO had the lowest RMSD of 0.084 Å, whereas sPPO versus mPPO had the highest RMSD of 0.119 Å. Modeling reveals that the N-terminal extensions neither adopt an ordered secondary structure, nor interfere with the PPO functional domain structure. Functional data reveals that all PPO proteins function well in plants, suggesting that the varying N-termini do not impact functional performance.

## Discussion

### Challenges in production of tag-free PPO protein

Transgenic maize, cotton, and soybean tolerant to PPO-inhibiting herbicides have been developed by constitutively expressing PPO targeted to the chloroplast via addition of a known CTP to PPO protein [7]. After the precursor polypeptide is imported into the chloroplast, the CTP is removed by stromal processing peptidase and then rapidly degraded by various peptidases [37, 38]. However, whether these transgenic CTPs are completely or partially processed *in vivo* must be experimentally determined. Both Edman degradation and mass spectrometry can be used for protein N-terminal determination. Typically, Edman requires more (>10 picomole) and higher purity (>70% purity) protein than mass spectrometry. Additionally, Edman does not work when a protein is N-terminally blocked through protein modifications such as acetylation. While the mass spectrometry method is very sensitive and robust, the availability of a peptidase which can produce a suitable size of N-terminal peptide through its digestion of a protein is crucial for success of mass spectrometry. Proteins can undergo degradation or chemical modifications during sample preparation, storage, or analysis, impacting the N-terminal sequence or structure. In the case of PPO proteins, N-terminal peptides generated by trypsin are either too short or too long for determination of sequence via mass spectrometry analysis. Therefore, alternative peptidases were needed for the mass spectrometry method. Additionally, isolation of highly pure PPO protein from plant tissue was extremely challenging due to its intrinsic propensity to aggregate, which also limited use of the Edman method. After multiple runs using optimized experimental conditions, results from Edman and mass spectrometry analyses demonstrate partial CTP processing in transgenic maize and soybean, resulting in variants with up to 13 CTP-derived amino acids retained at the N-terminus, whereas processing in cotton is complete. Incomplete processing of the CTP from precursor proteins targeted to the chloroplast is frequently observed in other transgenic crops and has not been found to impact functional activity [17, 18, 39]. One focus of the present study is to assess whether small differences in N-terminal amino acid sequence would impact PPO functional properties.

To conduct functional characterization, we planned to isolate these PPO variants without an affinity tag at large scale from heterologous bacterial sources. Screening of *E*. *coli* expression systems for PPO demonstrated a requirement for high concentrations of salt and detergent to solubilize PPO. However, high salt-containing buffers interfere with ion exchange chromatography, and high concentrations of detergent are incompatible with hydrophobic interaction chromatography. Further dilution of the soluble extracts is prohibitive at large scale, precluding these traditional purification methods for large scale PPO purification.

Because conventional chromatography methods were not feasible for large scale tag-free PPO production, we pivoted to testing a variety of affinity tags including His, SUMO, thioredoxin, Mistic and CL7/Im7. Each of these tags was integrated into an expression vector to produce a PPO-fusion protein that could use the corresponding tag affinity resin for purification. Notably, the CL7/Im7 tag allows one step purification [40]. The Mistic tag was developed for facilitating membrane protein production [41]. Ideally, these tags would be removed by an appropriate protease; however, PPO fusions were highly inefficient at protease-mediated tag cleavage for all systems screened. We postulate that inefficient cleavage is driven by one of two possibilities: either an incompatible buffer that inhibits protease activity or steric hindrance at the cleavage site precluding protease access. The high concentrations of salt and detergent needed for PPO isolation have not been tested for effect on protease activity, so this possibility remains unexplored. However, the association of PPO with detergent micelles suggests a reasonable likelihood for steric interference.

### Development of a novel method for active PPO protein production

As the approaches described above for large scale production of tag-free PPO were not successful, a His-tagged PPO construct was designed. In the His-tag affinity strategy, a histidine tag, often 6 residues in length, is covalently attached to a target protein at either the N- or C-terminus, and inclusion of these residues is generally shown to have a negligible effect on the native structure of the protein [42]. Indeed, over 16,000 reported protein structures determined by X-ray diffraction include a His-tag in SEQRES records [43]. Further, numerous studies have been conducted using His-tagged proteins, including, but not limited to, enzymatic activity assays [44–46], protein-protein interaction [47, 48], labeling [49, 50] and vaccine expression and purification [51]. In our hands, we have previously demonstrated that inclusion of a His-tag does not impact Mpp75Aa1 function or safety [52].

During this study, we found that a C-terminal 6xHis-tag reduced PPO expression in *E*. *coli*. However, His-tag placement at the N-terminus of PPO shows strong expression in our optimized system. We do not have a direct explanation for this observation, although we postulate that the native N-terminus of PPO may be prohibitive for overexpression in *E*. *coli*, which would be consistent with observations of low expression levels for tag-free PPO. By adding six histidine residues at the N-terminus, we may have changed the initiation amino acid sequence to a more favorable sequence for expression.

An additional challenge was to maintain PPO activity during and after purification. As discussed above, recombinant PPO requires high salt and detergent concentrations for isolation, but these components must be reduced for use in subsequent animal feeding studies, an essential component of protein safety assessment. After many attempts, L-arginine was found to be a critical factor in maintaining PPO activity under reduced salt and detergent concentrations during purification and concentration. We determined that salt can be removed and Thesit levels can be reduced to 0.1% in the presence of 56 mM L-arginine. This finding aligns with a previous report that arginine is useful for solubilization and purification of aggregate-prone membrane proteins [32]. Another challenge involved the high concentration of imidazole (> 0.15 M) needed to elute PPO protein from Ni-NTA affinity resin. At this imidazole concentration, PPO activity is negatively impacted. We screened alternative elution compounds and found L-histidine can substitute for imidazole without impacting PPO activity. Using this optimized His-tag purification methodology, we purified several PPO variants of varying N-terminal amino acid sequence.

### Demonstration that small N-terminal amino acid changes do not impact PPO protein function

Given the importance of functional characterization of introduced proteins for protein safety assessment [19–22, 53], several functional and biochemical properties of the various forms of PPO were evaluated following a previously proposed strategy [54]. First, these PPO proteins were subjected to amino acid sequence analysis by mass spectrometry. The results confirmed that each PPO variant produced from *E*. *coli* displayed the expected sequence. SDS-PAGE analysis also verified each variant migrated at the expected apparent molecular weight. The PPO variants display equivalent enzyme activity, within the CV of the assay variance itself as well as the PI, demonstrating that these modifications at the N-terminus of PPO do not interfere with its functional activity. These results are consistent with previous observations on recombinant *E*. *coli* PPO and human PPO performed elsewhere [11, 23]. We also tested whether PPO immunoreactivity was affected by N-terminal differences in sequence. Immunoreactive analysis verifies the protein intactness and distinguishes potential differences in immunoreactive properties (Raybould et al., 2013). Results show immunoreactive properties are similar among all variants, including tag-free PPO.

These sequence, functional, and structural analyses confirmed that these PPO variants are physicochemically and functionally equivalent. A regulatory protein safety study package can be conducted using one of these variants, and the safety data can be applied for other PPO variants to avoid repeated *de novo* safety studies for each respective PPO variants. Although experimental structural determination was not successful, comparison of the respective variants using three different *in silico* prediction algorithms were performed. The RMSD values of these predicted structures for all variants ranged between 0.18 Å and 0.39 Å, with an overall average RMSD of 0.24 Å. The ESMfold predicted variant structures exhibited the highest RMSD. ESMfold uses a large language model to fold proteins quickly for metagenome analysis, thus the structure prediction may not be as accurate as the more time intensive Alphafold. When comparing only the Alphafold ranked_0 structures of each variant, the RMSDs ranged from 0.02–0.04 Å, which indicates that the overall fold between the variants is conserved.

Recently, an experimentally determined structure of a PPO homolog (flavodoxin FldH) from *Fusobacterium mucleatum* revealed the location of the active site [55]. The experimentally determined FldH structure has a similar overall fold compared to the *in silico* predicted structural models of PPO, despite very low sequence identity (13%) and high RMSD (11 Å) value. The FldH active site is located under the helical cap domain on the opposite end from the N-termini. In the PPO predicted structure, this cap domain seems to be in an alternate conformation, however still distant from the N-terminus. Due to the spatial separation of the termini from the active site, along with the functional characterization described herein, additions to the N-termini are unlikely to interfere with the catalytic activity of PPO.

### Challenges overcame to produce 200 grams of active PPO protein and formulate PPO protein at >75 mg/mL

A process was developed to purify ~70 grams of active PPO in a single day, and >200 grams were produced by repeating this process. Under this process, batch mode was used for binding of PPO to Ni-NTA resin and then transferred to a column for purifying PPO from impurities. Batch mode allows for PPO to quickly interact with more functional affinity groups of Ni-NTA resin while column chromatography is more robust for quickly enhancing purity. Endotoxins are lipopolysaccharides produced in *E*. *coli*, which can induce inflammation and fever as an immune response in animals. Removal of endotoxins can be a challenging task, especially for purification of a membrane associated protein. In this process, endotoxin removal was achieved using a large volume of 1% Thesit-containing buffer to wash the protein bound resin.

The protein produced must be amenable to concentration at high levels for animal toxicity studies using animal-friendly buffers. The latter constraint limits the use of detergents, high/low pH, high concentration of salt, etc. for protein formulation/concentration. It is known that arginine is a Generally Recognized as Safe substance by the US Food and Drug Administration [56]. Arginine has been used to enhance protein refolding and solubility for various proteins, including membrane proteins, by stabilizing the protein, suppressing aggregation, reducing the viscosity of high concentration protein formulations and reducing nonspecific surface adsorption [32, 57]. Enhancing protein solubility through the addition of arginine to refolding solutions appears to interrupt disulfide bond formation and to maintain the solubility of partially folded polypeptides that have free thiol groups [58]. On the other hand, arginine can also facilitate *in vitro* refolding of disulfide-free proteins. This occurs possibly through suppression of protein aggregation by the arginine side chain (the guanidinium group), but not through directly impacting the protein refolding reaction [58]. However, ionic arginine at or below neutral pH (arginine pI: 10.7) may interfere with the refolding of proteins that require low ionic strength during refolding [58]. After many tests aligning these previous findings [32, 57, 58], a simple buffer including 5 mM sodium phosphate, 28 mM arginine, 0.1 mM FMN and pH 10.3 was developed and all components are animal feeding friendly. PPO was able to be concentrated to >75 mg/mL without loss of enzyme activity.

## Conclusions

The amino acid sequences of PPO variants expressed in transgenic cotton, maize and soybean were found to vary due to differential processing of the CTP attached to each variant. Traditionally, each variant would be expressed in a heterologous system, purified at large scale, and subjected to a full protein safety assessment prior to commercialization. However, due to the intrinsic technical difficulty in expression and purification of active PPO at this large scale, we suggest that a single representative safety study package using the mature PPO (named as PPO in Fig 1C), can be leveraged for each of these PPO variants, as we showed here through bridging studies that they have similar biochemical properties, including functional activity. In addition, structural modeling shows that these small differences at the N-termini should not alter the mature structure of the PPO protein where the active site is located. Taken together, the structural modeling and functional characterization demonstrate that N-terminal extensions with His-tag inclusion or the incomplete processing of the CTP sequence does not affect PPO function.

It has been observed that other proteins including DMO and Cry1A.105 with minor sequence differences at the N-terminus do not impact activity nor results of safety studies [17, 18]. A bridging approach developed for an animal toxicity study, confirming that biotech trait proteins with slightly different N-termini have equivalent physiochemical and functional properties, has been accepted by EFSA to approve a GM corn product [59]. By using this bridging approach for regulatory safety assessments of proteins with small differences in their N-terminal sequences, up to 260 mice can be spared for each new protein variant by leveraging previous safety studies.

We propose that a safety assessment using the 200 g of His-tagged and highly active PPO protein will be a representative study for PPO variants that demonstrate physiochemical and functional equivalence.

## Supporting information

S1 Raw images The original images for Fig 1B, Fig 2 and Fig 3. (PDF)

S1 Table PPO variant amino acid sequences. Supplementary data include plant produced and *E*. *coli*-produced PPO variant sequences.
(DOCX)

S2 Table Conditions screened for large scale tag-free PPO production. (DOCX)

S3 Table Optimized conditions for large scale PPO production. (DOCX)

S4 Table Individual assay data and 95% prediction interval of the PPO functional activity for one future assay. (DOCX)

S5 Table Raw data from PPO activity assays for Table 2. (DOCX)

S1 File Identity of the N-terminal peptide of the plant-produced PPO. Supplementary data for Fig 1C include tandem mass spectrometry (MS/MS) spectra of the N-terminal peptide for maize- and soybean-produced PPO, along with Edman sequencing data for the N-terminus of the cotton-produced PPO.
(PDF)

S2 File SDS PAGE analysis of PPO variants produced from *E*. *coli*. Supplementary data include molecular weight determination and purity analysis for Table 2. The SDS-PAGE gel was analyzed by a Bio-Rad GS-900 Calibrated Densitometer using Bio-Rad Image Lab Security Edition Software version 6.1.0 build 7.
(PDF)

S3 File Identity of *E*. *coli*-produced PPO. Supplementary data for Table 2 include tandem mass spectrometry (MS/MS) spectra of the N-terminal and C-terminal peptides, as well as sequence coverage for *E*. *coli*-produced PPO variants.
(PDF)
