# Supplementary material for: Production and characterization of homologous protoporphyrinogen IX oxidase (PPO) proteins: Evidence that small N-terminal amino acid changes do not impact protein function
Source: PLoS One. 2024 Sep 26;19(9):e0311049. doi: 10.1371/journal.pone.0311049 (PMC11426539; doi:10.1371/journal.pone.0311049)
Supplement: S1 Raw images — (PDF) [file pone.0311049.s001.pdf]

Fig 1. Original image for lanes 1, 2 and 3 of Fig 1B

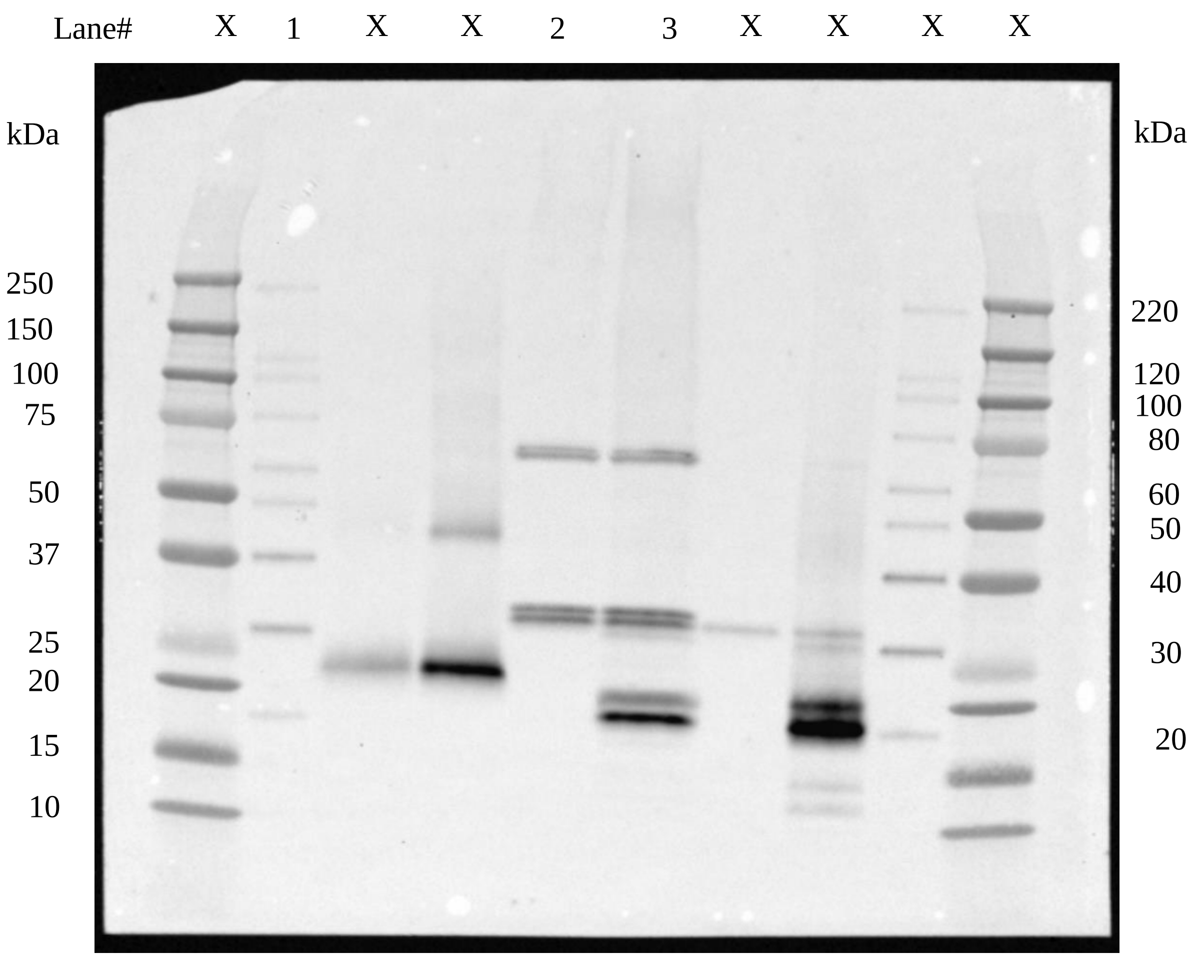

Western blot analysis of PPO protein extracted from wild-type and PPO-transgenic maize. Image was captured by a Bio-Rad ChemiDoc Touch Imaging System using Bio-Rad Image Lab Touch Software version 2.2.0.08.

Fig 1. Original image for lanes 4, 5 and 6 of Fig 1B

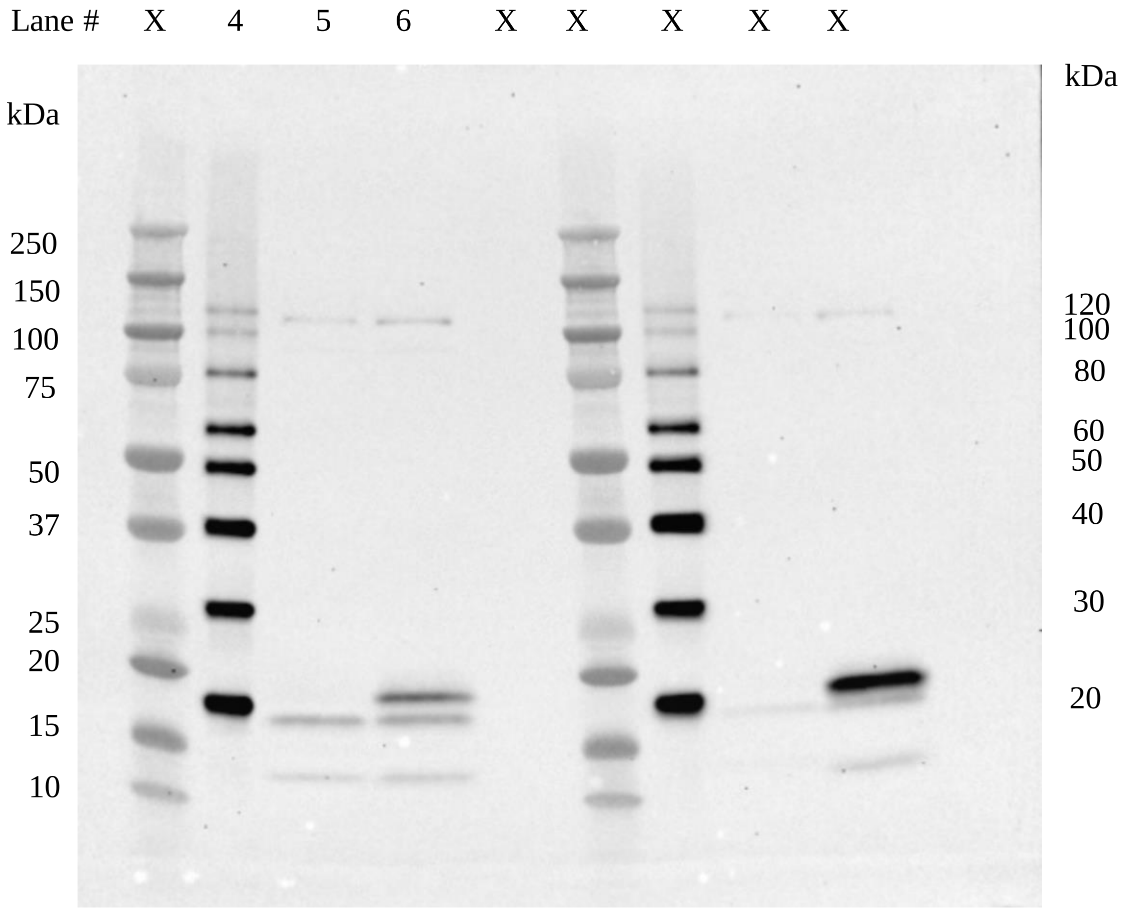

Western blot analysis of PPO protein extracted from wild-type and PPO-transgenic cotton. Image was captured by a Bio-Rad ChemiDoc Touch Imaging System using Bio-Rad Image Lab Touch Software version 2.2.0.08.

Fig 1. Original image for lanes 7, 8 and 9 of Fig 1B

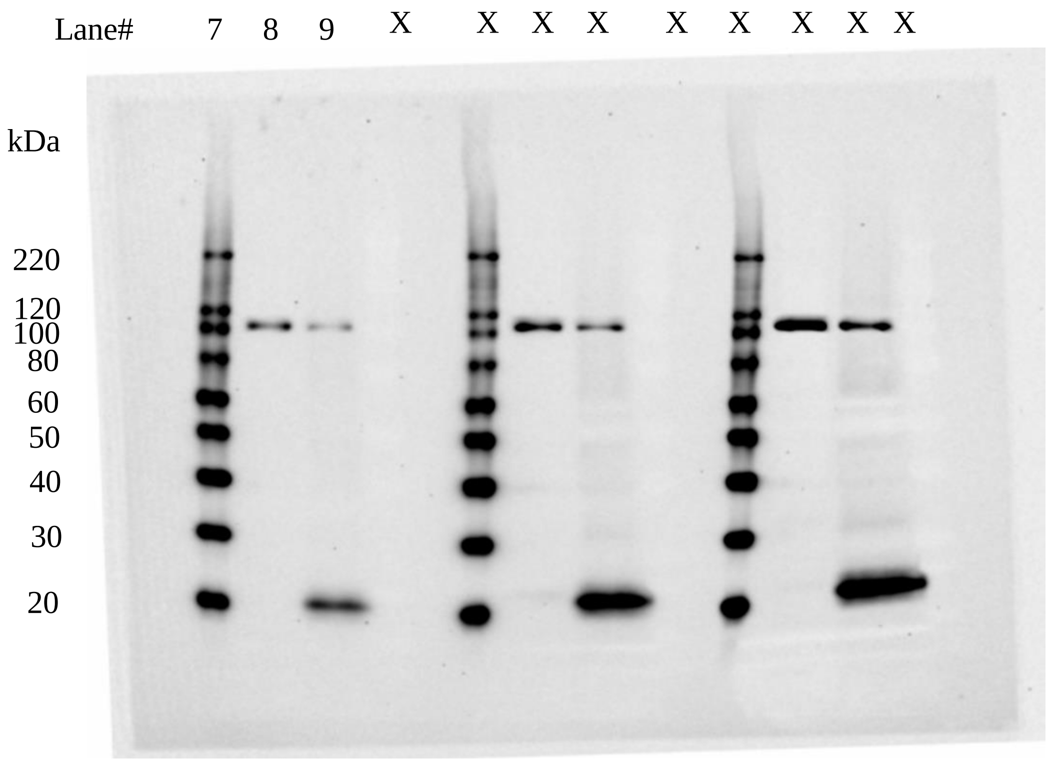

Western blot analysis of PPO protein extracted from wild-type and PPO-transgenic soybean. Image was captured by a Bio-Rad ChemiDoc Touch Imaging System using Bio-Rad Image Lab Touch Software version 2.2.0.08.

Fig 2. Original image for Fig 2

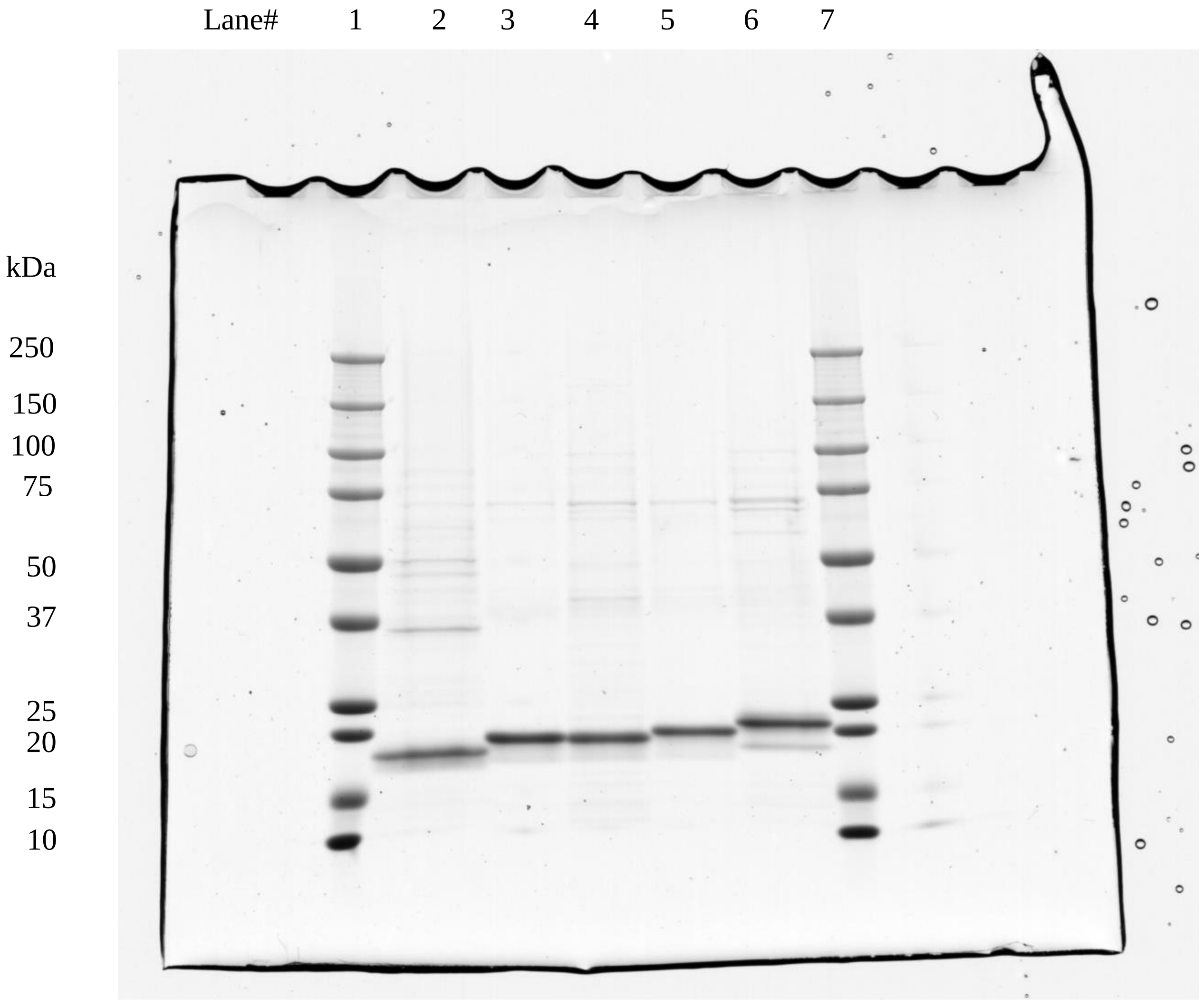

SDS PAGE analysis of PPO protein produced from *E. coli*. Image was captured by a Bio-Rad GS-900 Calibrated Densitometer using Bio-Rad Image Lab Security Edition Software version 6.1.0 build 7.

Fig 3. Original image for Fig 3

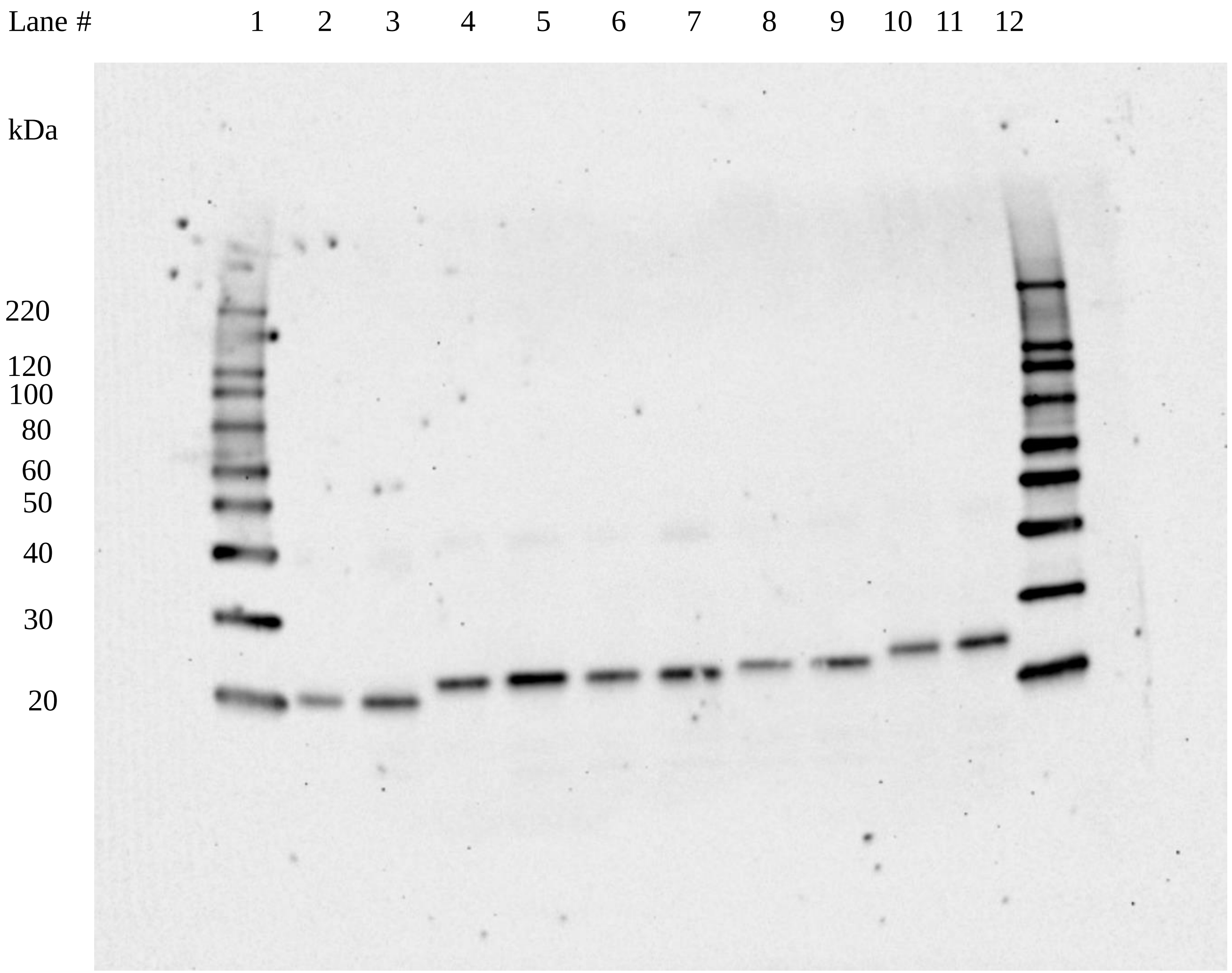

Western blot analysis of PPO protein produced from *E. coli.* Image was captured by a Bio-Rad ChemiDoc Touch Imaging System using Bio-Rad Image Lab Touch Software version 2.2.0.08.
