# Supplementary material for: Production and characterization of homologous protoporphyrinogen IX oxidase (PPO) proteins: Evidence that small N-terminal amino acid changes do not impact protein function
Source: PLoS One. 2024 Sep 26;19(9):e0311049. doi: 10.1371/journal.pone.0311049 (PMC11426539; doi:10.1371/journal.pone.0311049)
Supplement: S1 Table — Supplementary data include plant produced and E. coli-produced PPO variant sequences. (DOCX) [file pone.0311049.s002.docx]

**S1 Table. PPO variant amino acid sequences**

| Plant Produced | | *E. coli* Produced | |
| --- | --- | --- | --- |
| Maize PPO | KALVLYSTRDGQTHAIASYIASCMKEKAECDVIDLTHGEHVNLTQYDQVLIGASIRYGHFNAVLDKFIKRNVDQLNNMPSAFFCVNLTARKPEKRTPQTNPYVRKFLLATPWQPALCGVFAGALRYPRYRWIDKVMIQLIMRMTGGETDTSKEVEYTDWEQVKKFAEDFAKLSYKKAL | PPO | MHHHHHHKALVLYSTRDGQTHAIASYIASCMKEKAECDVIDLTHGEHVNLTQYDQVLIGASIRYGHFNAVLDKFIKRNVDQLNNMPSAFFCVNLTARKPEKRTPQTNPYVRKFLLATPWQPALCGVFAGALRYPRYRWIDKVMIQLIMRMTGGETDTSKEVEYTDWEQVKKFAEDFAKLSYKKAL |
|  | TRRLDHRPFVVRCKALVLYSTRDGQTHAIASYIASCMKEKAECDVIDLTHGEHVNLTQYDQVLIGASIRYGHFNAVLDKFIKRNVDQLNNMPSAFFCVNLTARKPEKRTPQTNPYVRKFLLATPWQPALCGVFAGALRYPRYRWIDKVMIQLIMRMTGGETDTSKEVEYTDWEQVKKFAEDFAKLSYKKAL | mPPO | MHHHHHHTRRLDHRPFVVRCKALVLYSTRDGQTHAIASYIASCMKEKAECDVIDLTHGEHVNLTQYDQVLIGASIRYGHFNAVLDKFIKRNVDQLNNMPSAFFCVNLTARKPEKRTPQTNPYVRKFLLATPWQPALCGVFAGALRYPRYRWIDKVMIQLIMRMTGGETDTSKEVEYTDWEQVKKFAEDFAKLSYKKAL |
| Soy PPO | DASKALVLYSTRDGQTHAIASYIASCMKEKAECDVIDLTHGEHVNLTQYDQVLIGASIRYGHFNAVLDKFIKRNVDQLNNMPSAFFCVNLTARKPEKRTPQTNPYVRKFLLATPWQPALCGVFAGALRYPRYRWIDKVMIQLIMRMTGGETDTSKEVEYTDWEQVKKFAEDFAKLSYKKAL | sPPO | MHHHHHHDASKALVLYSTRDGQTHAIASYIASCMKEKAECDVIDLTHGEHVNLTQYDQVLIGASIRYGHFNAVLDKFIKRNVDQLNNMPSAFFCVNLTARKPEKRTPQTNPYVRKFLLATPWQPALCGVFAGALRYPRYRWIDKVMIQLIMRMTGGETDTSKEVEYTDWEQVKKFAEDFAKLSYKKAL |
| Cotton PPO | MKALVLYSTRDGQTHAIASYIASCMKEKAECDVIDLTHGEHVNLTQYDQVLIGASIRYGHFNAVLDKFIKRNVDQLNNMPSAFFCVNLTARKPEKRTPQTNPYVRKFLLATPWQPALCGVFAGALRYPRYRWIDKVMIQLIMRMTGGETDTSKEVEYTDWEQVKKFAEDFAKLSYKKAL | cPPO | MHHHHHHMKALVLYSTRDGQTHAIASYIASCMKEKAECDVIDLTHGEHVNLTQYDQVLIGASIRYGHFNAVLDKFIKRNVDQLNNMPSAFFCVNLTARKPEKRTPQTNPYVRKFLLATPWQPALCGVFAGALRYPRYRWIDKVMIQLIMRMTGGETDTSKEVEYTDWEQVKKFAEDFAKLSYKKAL |
|  |  | Tag-free PPO | MKALVLYSTRDGQTHAIASYIASCMKEKAECDVIDLTHGEHVNLTQYDQVLIGASIRYGHFNAVLDKFIKRNVDQLNNMPSAFFCVNLTARKPEKRTPQTNPYVRKFLLATPWQPALCGVFAGALRYPRYRWIDKVMIQLIMRMTGGETDTSKEVEYTDWEQVKKFAEDFAKLSYKKAL |
