# Supplementary material for: Production and characterization of homologous protoporphyrinogen IX oxidase (PPO) proteins: Evidence that small N-terminal amino acid changes do not impact protein function
Source: PLoS One. 2024 Sep 26;19(9):e0311049. doi: 10.1371/journal.pone.0311049 (PMC11426539; doi:10.1371/journal.pone.0311049)
Supplement: S2 Table — (DOCX) [file pone.0311049.s003.docx]

**S2 Table. Conditions screened for large scale tag-free PPO production**

| **Construct** | **Expression** | **Solubility with detergent** | **Challenges** | **Conditions** |
| --- | --- | --- | --- | --- |
| Tag-free | Medium | Medium | Conventional chromatography methods (ion exchange, hydrophobic interaction, ceramic hydroxyapatite) were ineffective | **Four cell lines:**  BL21(*DE3*), BL21(*DE3*)RIPL, C41(*DE3*) and C43(*DE3*)  **Three media:**  Luria Broth, Terrific Broth and auto induction medium  **Three temperatures**:  16, 20 and 30 °C  **11 detergents**:  Triple [10% lauryl maltose neopentyl glycol (LMNG) /1.5% soybean L-α-phosphatidylcholine (PC) /0.5% Cholesteryl hemisuccinate (CHS)], Triton-X-100 (up to 1%), Thesit (up to 2%), Tween 20 (up to 0.5%), Tween 80 (up to 0.3%), N-dodecyl-beta-maltoside (up 1%), n-octyl glucoside (up to 2%), sodium cholate (up to1%), LMNG alone and TritonX-100, CHAPS  **Five base buffers:**  CAPS (N-cyclohexyl-3-aminopropanesulfonic acid) pH 11.0, HEPES pH 7.5 & pH 8.0, Tris-HCl pH 8.0, sodium acetate pH 5, and phosphate buffer pH7.4  **Buffer Additives:**  NaCl, L-Arginine, Arginine-HCl, Histidine, Glutamine, glycerol, DTT and FMN |
| Thioredoxin-PPO | Medium | Medium | No tag cleavage with protease |  |
| 10xHis-TVMV-PPO | Medium | Medium | No tag cleavage with TVMV protease |  |
| SUMO-PPO | Medium | 80 to 90% | <5% tag cleavage with SUMO protease |  |
| Mistic-PPO | High | Insoluble | No tag cleavage with enterokinase protease |  |
|  |  | Soluble in 6 M urea | < 5% tag cleavage |  |
| CL7/Im7-PPO | High | Soluble | < 5% tag cleavage |  |
