# Supplementary material for: Production and characterization of homologous protoporphyrinogen IX oxidase (PPO) proteins: Evidence that small N-terminal amino acid changes do not impact protein function
Source: PLoS One. 2024 Sep 26;19(9):e0311049. doi: 10.1371/journal.pone.0311049 (PMC11426539; doi:10.1371/journal.pone.0311049)
Supplement: S3 Table — (DOCX) [file pone.0311049.s004.docx]

**S3 Table. Optimized conditions for large scale PPO production**

| **Step** | **Details** |
| --- | --- |
| **Construct** | The N-terminal 6xHis tagged PPO was cloned into the pET24 vector |
| **Expression conditions** | The plasmid was transformed into BL21(DE3) E. coli, and the cells were grown in auto-induction media with 50 µg/mL kanamycin at 37°C for 4 hours, followed by overnight induction at 16°C |
| **Extraction of protein** | The cells were lysed using a microfluidizer and proteins were extracted with buffer: 50 mM HEPES, 300 mM NaCl, 2 mM MgCl2, 10 mM imidazole, 0.1 mM FMN, 10% glycerol, 2% Thesit, benzonase (100 U/mL), lysozyme (100 µg/mL), 2 mM benzamidine, complete protease inhibitor (1 tablet/100 mL), pH 7.5, at 20 L/kg cell paste |
| **Ni-NTA resin chromatography** | PPO binding to the Ni-NTA resin was performed in batch mode, followed by washing and elution in column mode (2.5 kg cell paste/8 L Ni-NTA resin) |
| **Wash** | The column was washed with 10 column volumes (CV) of Wash I: 50 mM HEPES, 50 mM NaCl, 15 mM imidazole, 0.1 mM FMN, 20% glycerol, 1% Thesit, pH 8.0.  Five CV of Wash II: 50 mM HEPES, 50 mM NaCl, 20 mM imidazole, 0.1 mM FMN, 20% glycerol, 0.1% Thesit, pH 8.0 |
| **Elution** | PPO protein was eluted with 5 CV of Elution Buffer: 11 mM phosphate (pH 7.4-7.6), 135 mM NaCl, 2.7 mM KCl, 56 mM L-arginine, 80 mM L-histidine, 60 mM imidazole, 0.1 mM FMN, 20% glycerol, 0.1% Thesit, pH 9.0 |
| **Concentration** | The eluted PPO protein was buffer-exchanged for 10 turnovers with Buffer: 5 mM monosodium phosphate, 28 mM L-arginine, 0.1 mM FMN, pH 10.3, then concentrated to the expected concentration |
