# Supplementary material for: Production and characterization of homologous protoporphyrinogen IX oxidase (PPO) proteins: Evidence that small N-terminal amino acid changes do not impact protein function
Source: PLoS One. 2024 Sep 26;19(9):e0311049. doi: 10.1371/journal.pone.0311049 (PMC11426539; doi:10.1371/journal.pone.0311049)
Supplement: S4 Table — (DOCX) [file pone.0311049.s005.docx]

**Prediction intervals for PPO functional activity assay acceptance criteria**

The data used for predicting interval (PI) were generated and calculated according to the experimental design and statistical analysis described by Urquhart et al. (2015). The calculation was validated using SAS Software (Release 9.4, 2012). The activity was measured at room temperature as described in the Materials and Methods section.

**S4 Table. Individual assay data and 95% prediction interval for the PPO functional activity for one future assay**

| **Assay Number** | **Replicates of Functional Activity Assay**  **(nmol min^-1^ mg^-1^)** | **Mean of Functional Activity of PPO Protein^1^**  **(nmol min^-1^ mg^-1^)** |
| --- | --- | --- |
| 1 | 162.1, 146.0 | 154 |
| 2 | 195.0, 184.7 | 190 |
| 3 | 185.8, 180.6 | 183 |
| 4 | 233.0, 218.7 | 226 |
| 5 | 177.4, 150.2 | 164 |
| 6 | 252.7, 250.0 | 251 |
| 7 | 197.3, 234.0 | 216 |
| 8 | 210.6, 198.0 | 204 |
| **Mean** |  | 198 |
| **Standard Deviation** |  | 32 |
| **95% Prediction Interval** |  | 117 – 280 |

**^1^** The values in the table represent the mean of 8 assays (n=8), with each assay performed in duplicate. Data were generated by three analysts using the *E.coli*-produced His-tagged mature form PPO (PPO). With 95% confidence, the mean from the next single assay will fall within the stated interval. Values for mean have been rounded to nearest whole number for tabulation.

**References:**

SAS (2012). Software Release 9.4 (TS1M4). Cary, North Carolina, Copyright 2002-2012 by SAS Institute, Inc.

Urquhart W, Mueller GM, Carleton S, Song Z, Perez T, et al. 2015. A novel method of demonstrating the molecular and functional equivalence between in vitro and plant-produced double-stranded RNA. *Regulatory Toxicology and Pharmacology* 73: 607-12
