# Supplementary material for: Production and characterization of homologous protoporphyrinogen IX oxidase (PPO) proteins: Evidence that small N-terminal amino acid changes do not impact protein function
Source: PLoS One. 2024 Sep 26;19(9):e0311049. doi: 10.1371/journal.pone.0311049 (PMC11426539; doi:10.1371/journal.pone.0311049)
Supplement: S5 Table — (DOCX) [file pone.0311049.s006.docx]

**Raw data from PPO activity assays for Table 2**

| **Activity^1^**  (nmol min^-1^ mg^-1^) | | | | | |
| --- | --- | --- | --- | --- | --- |
|  | **Tag-free PPO** | **PPO** | **mPPO** | **cPPO** | **sPPO** |
|  | 218 | 196 | 333 | 224 | 213 |
|  | 195 | 253 | 257 | 205 | 183 |
|  | 192 | 189 | 229 | 198 | 207 |
|  | 169 | 259 | 253 | 228 | 199 |
|  |  | 89 | 268 |  |  |
|  |  | 263 | 271 |  |  |
|  |  | 226 | 250 |  |  |
|  |  | 228 | 266 |  |  |
|  |  | 227 |  |  |  |
|  |  | 234 |  |  |  |
|  |  | 235 |  |  |  |
|  |  | 221 |  |  |  |
|  |  | 215 |  |  |  |
|  |  | 236 |  |  |  |
|  |  | 220 |  |  |  |
|  |  | 209 |  |  |  |
|  |  | 224 |  |  |  |
|  |  | 212 |  |  |  |
|  |  | 219 |  |  |  |
| **Mean** | 193 | 219 | 266 | 214 | 200 |
| **Standard Deviation** | 20 | 37 | 30 | 14 | 13 |

^1^Reported values are replicates used to calculate the mean and standard deviation for each PPO variant activity. All assays were run using PPO as a reference, leading to an increased number of replicates for PPO relative to the other variants. For mPPO, an additional assay was performed. Values have been rounded to nearest whole number for tabulation
