# Supplementary material for: Production and characterization of homologous protoporphyrinogen IX oxidase (PPO) proteins: Evidence that small N-terminal amino acid changes do not impact protein function
Source: PLoS One. 2024 Sep 26;19(9):e0311049. doi: 10.1371/journal.pone.0311049 (PMC11426539; doi:10.1371/journal.pone.0311049)
Supplement: S1 File — Supplementary data for Fig 1C include tandem mass spectrometry (MS/MS) spectra of the N-terminal peptide for maize- and soybean-produced PPO, along with Edman sequencing data for the N-terminus of the cotton-produced PPO. (PDF) [file pone.0311049.s007.pdf]

Maize PPO N-term

Mascot Search Results

Peptide View

MS/MS Fragmentation of **KALVLY**
Found in **>gi|MaizePPO_** in **MaizePPO**, MaizePPO_

Match to Query 7827: 705.440902 from(353.727727,2+) intensity(1904278728.5000) rtinseconds(1923.114668) index(19645)
Title: MaizePPO.20887.20887.2
Data file C:\Users\EKYSC\Desktop\MaizePPO.raw

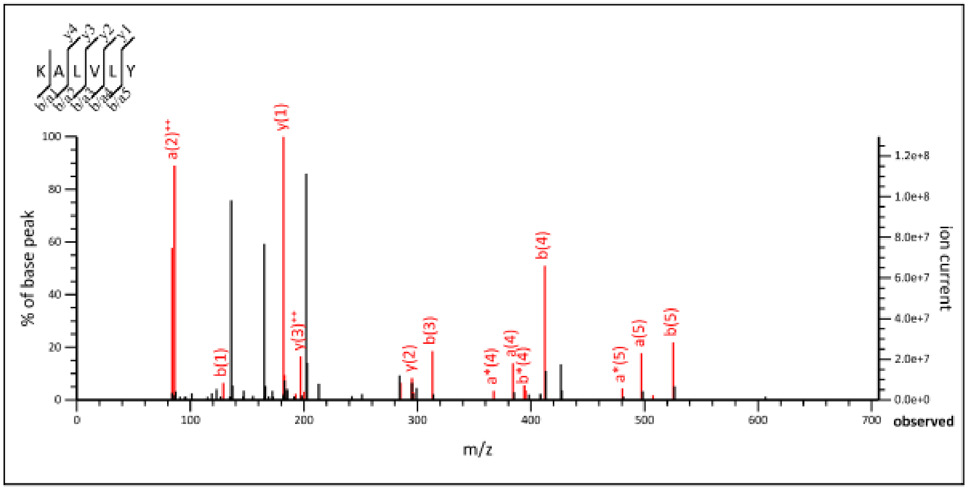

Label all possible matches ○ Label matches used for scoring ◉

Monoisotopic mass of neutral peptide Mr(calc): 705.4425
Fixed modifications: Carbamidomethyl (C) (apply to specified residues or termini only)
Ions Score: 30 **Expect:** 0.00089
Matches : 21/50 fragment ions using 38 most intense peaks (help)

#	a	a++	a*	a*++	b	b++	b*	b*++	Seq.	y	y++	#
1	101.1073	51.0573	84.0808	42.5440	***129.1022***	65.0548	112.0757	56.5415	K			6
2	172.1444	**86.5759**	155.1179	78.0626	***200.1394***	100.5733	183.1128	92.0600	A	578.3548	289.6811	5
3	**285.2285**	143.1179	268.2020	134.6046	***313.2234***	157.1153	296.1969	148.6021	L	**507.3177**	254.1625	4
4	**384.2969**	**192.6521**	367.2704	184.1388	***412.2918***	206.6496	395.2653	198.1363	V	**394.2336**	197.6205	3
5	**497.3810**	249.1941	480.3544	240.6809	***525.3759***	263.1916	508.3493	254.6783	L	**295.1652**	148.0863	2
6									Y	**182.0812**	91.5442	1

Mascot Search Results

Peptide View

MS/MS Fragmentation of **TRRLDHRPF**
Found in **>gi|MaizePPO** in **MaizePPO**, MaizePPO

Match to Query 28990: 1196.651964 from(599.333258,2+) intensity(53223915.5000) rtinseconds(1054.867749) index(10771)
Title: MaizePPO_.11449.11449.2
Data file C:\Users\EKYSC\Desktop\MaizePPO_.raw

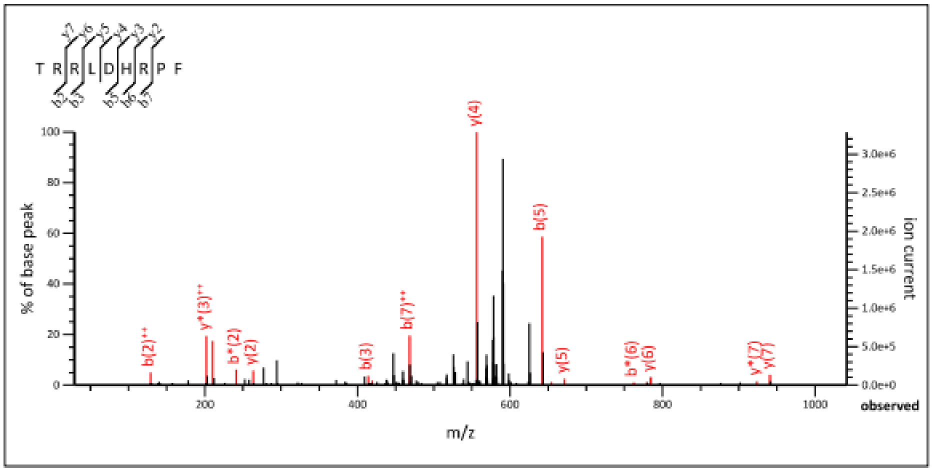

Label all possible matches ○ Label matches used for scoring ◉

Monoisotopic mass of neutral peptide Mr(calc): 1196.6527
Fixed modifications: Carbamidomethyl (C) (apply to specified residues or termini only)
Ions Score: 33 **Expect:** 0.0005
Matches : 17/88 fragment ions using 33 most intense peaks (help)

#	a	a++	a*	a*++	b	b++	b*	b*++	Seq.	y	y++	y*	y*++	#
1	74.0600	37.5337			102.0550	51.5311			T					9
2	230.1612	115.5842	213.1346	107.0709	258.1561	**129.5817**	241.1295	121.0684	R	1096.6123	548.8098	1079.5857	540.2965	8
3	386.2623	193.6348	369.2357	185.1215	**414.2572**	207.6322	397.2306	199.1190	R	***940.5112***	470.7592	923.4846	462.2459	7
4	499.3463	250.1768	482.3198	241.6635	527.3412	264.1743	510.3147	255.6610	L	***784.4100***	392.7087	767.3835	384.1954	6
5	614.3733	307.6903	597.3467	299.1770	**642.3682**	321.6877	625.3416	313.1745	D	***671.3260***	336.1666	654.2994	327.6534	5
6	751.4322	376.2197	734.4056	367.7065	**779.4271**	390.2172	762.4005	381.7039	H	***556.2990***	278.6532	539.2725	270.1399	4
7	907.5333	454.2703	890.5067	445.7570	935.5282	**468.2677**	918.5017	459.7545	R	***419.2401***	210.1237	402.2136	201.6104	3
8	1004.5861	502.7967	987.5595	494.2834	1032.5810	516.7941	1015.5544	508.2808	P	***263.1390***	132.0731			2
9									F	166.0863	83.5468			1

Cotton PPO N-term

[Sequence Analysis]

Data Acquired	:	4/1/2022 3:10:21 AM
Data Processed	:	4/6/2022 1:47:32 PM
Reactor	:	3
Number of Cycles	:	11
Sequence Schedule	:	PVDF9-3G.SCH
Sample Name	:	2020220331ekxmp PPO
Sample Amount(pmol)	:	10.0
Sample ID	:	PPO
Operator Name	:	Prince Asare
Data File	:	2020220331ekxmp PPO
	:	2020 - 1-1131/1-2201-5 - 2020220331ekxmp PPO_D01.lcd
	:	2020 - 1-1131/1-2202-5 - 2020220331ekxmp PPO_D02.lcd
	:	2020 - 1-1131/1-2203-5 - 2020220331ekxmp PPO_D03.lcd
	:	2020 - 1-1131/1-2204-5 - 2020220331ekxmp PPO_D04.lcd
	:	2020 - 1-1131/1-2205-5 - 2020220331ekxmp PPO_D05.lcd
	:	2020 - 1-1131/1-2206-5 - 2020220331ekxmp PPO_D06.lcd
	:	2020 - 1-1131/1-2207-5 - 2020220331ekxmp PPO_D07.lcd
	:	2020 - 1-1131/1-2208-5 - 2020220331ekxmp PPO_D08.lcd
	:	2020 - 1-1131/1-2209-5 - 2020220331ekxmp PPO_D09.lcd
	:	2020 - 1-1131/1-2210-5 - 2020220331ekxmp PPO_D10.lcd
Start Number	:	1
Method File	:	2020220331ekxmp PPO.lcm
Batch File	:	2020220331ekxmp PPO.lcb
Number of Analyses	:	11 / 11
Standard File	:	2020 - 1-1108/1-2161-3 - 20220330ekxmp1PTH_D01.lcd
Data Comment		

[Sequence]

Met Lys Ala Leu Val Leu Tyr Ser Thr Pro

[Estimated Sequence]

	1	2	3	4	5	6	7	8	9	10
1st	Met	Lys	Ala	Leu	Val	Leu	Tyr	Pro	Thr	Pro
2nd	Gly	Pro	Pro	Met	Pro	Gln	Asp	Ser	Asp	Arg
3rd	Thr	Val	Val	Pro	Thr	Pro	Pro	Arg	Pro	Gln
4th	Asp	Gln	Gln	Glu	Glu	Arg	Thr	Ala	Arg	Lys
Reliability(%)	20.6	46.8	87.3	30.8	75.3	25.4	18.6	11.1	9.6	13.9

[Evaluated Value]

	1	2	3	4	5	6	7	8	9	10
Asp	12.99	0.85	3.89	0.88	0.87	0.75	25.51	0.59	17.44	0.83
Glu	0.67	3.52	1.55	4.35	3.49	2.45	1.91	1.19	2.73	0.71
Asn	0.80	2.78	1.85	1.43	2.11	1.23	2.49	1.49	2.00	0.83
Ser	12.50	0.63	0.91	0.93	1.36	0.86	0.93	8.37	0.78	0.86
Thr	13.78	0.44	0.81	0.97	6.67	0.72	4.76	0.73	17.56	0.71
Gln	0.69	5.07	4.57	4.32	3.21	7.13	0.80	1.99	2.93	2.16
Gly	29.23	0.78	0.99	3.86	0.88	0.84	0.95	0.89	0.91	0.83
His	0.96	1.23	0.91	0.86	0.90	0.91	0.95	0.89	0.90	0.83
Ala	1.79	0.86	61.56	0.35	0.84	4.05	1.14	2.47	0.87	0.85
Arg	3.70	0.70	1.11	4.33	0.82	4.64	2.88	4.54	3.68	2.48
Tyr	2.42	0.80	0.89	2.02	1.46	2.19	45.29	0.53	0.76	0.87
Pro	0.53	9.12	5.56	5.73	7.81	5.89	6.70	9.99	6.69	3.63
Met	65.97	0.10	0.29	9.09	0.38	0.65	0.86	1.29	0.87	0.78
Val	0.25	8.92	4.97	0.65	63.22	0.41	0.86	1.96	0.86	0.86
Trp	3.09	0.08	0.70	0.59	0.96	1.01	0.87	0.88	0.85	0.86
Lys	0.12	52.81	0.27	0.92	1.57	0.82	0.92	0.97	0.78	0.93
Phe	0.92	2.51	0.93	0.82	0.89	0.93	0.93	1.90	0.90	0.91
Ile	0.95	1.15	1.46	0.76	1.28	1.32	0.97	0.89	0.97	0.87
Leu	0.79	3.44	4.14	31.00	0.47	21.89	0.56	0.87	2.74	0.77

Soy PPO N-term

Mascot Search Results

Peptide View

MS/MS Fragmentation of **DASKALVLYSTR**
Found in **SoybeanPPO** in **SoybeanPPO**, SoybeanPPO

Match to Query 2528: 1322.719992 from(441.913940,3+) intensity(21131287.5000) rtinseconds(1480.963416) index(6010)
Title: SoybeanPPO.9446.9446.3
Data file C:\Users\EKYSC\Desktop\New folder\SoybeanPPO.raw

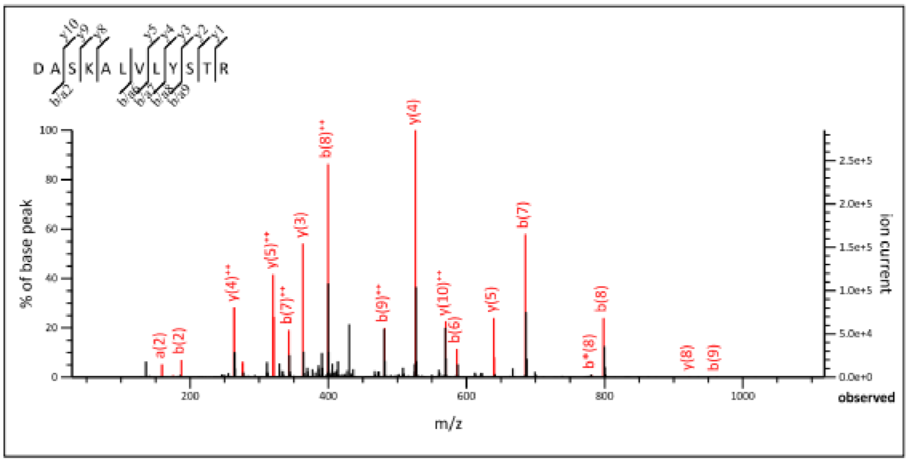

Label all possible matches ○ Label matches used for scoring ◉

```
Monoisotopic mass of neutral peptide Mr(calc): 1322.7194
Fixed modifications: Carbamidomethyl (C) (apply to specified residues or termini only)
Ions Score: 32  Expect: 0.0007
Matches : 23/120 fragment ions using 45 most intense peaks   (help)
```

#	a	a++	a*	a*++	b	b++	b*	b*++	Seq.	y	y++	y*	y*++	#
1	88.0393	44.5233			116.0342	58.5207			D					12
2	159.0764	80.0418			***187.0713***	94.0393			A	1208.6997	604.8535	1191.6732	596.3402	11
3	246.1084	123.5579			274.1034	137.5553			S	1137.6626	**569.3350**	1120.6361	560.8217	10
4	374.2034	187.6053	357.1769	179.0921	402.1983	201.6028	385.1718	193.0895	K	1050.6306	**525.8189**	1033.6041	517.3057	9
5	445.2405	223.1239	428.2140	214.6106	473.2354	237.1214	456.2089	228.6081	A	***922.5356***	461.7715	905.5091	453.2582	8
6	558.3246	279.6659	541.2980	271.1527	***586.3195***	293.6634	569.2930	285.1501	L	851.4985	426.2529	834.4720	417.7396	7
7	657.3930	329.2001	640.3665	320.6869	***685.3879***	**343.1976**	668.3614	334.6843	V	738.4145	369.7109	721.3879	361.1976	6
8	770.4771	385.7422	753.4505	377.2289	***798.4720***	**399.7396**	781.4454	391.2264	L	***639.3461***	**320.1767**	622.3195	311.6634	5
9	933.5404	467.2738	916.5138	458.7606	***961.5353***	**481.2713**	944.5088	472.7580	Y	***526.2620***	**263.6346**	509.2354	255.1214	4
10	1020.5724	510.7898	1003.5459	502.2766	1048.5673	524.7873	1031.5408	516.2740	S	***363.1987***	182.1030	346.1721	173.5897	3
11	1121.6201	561.3137	1104.5936	552.8004	1149.6150	575.3111	1132.5885	566.7979	T	***276.1666***	138.5870	259.1401	130.0737	2
12									R	***175.1190***	88.0631	158.0924	79.5498	1
