# Supplementary material for: Production and characterization of homologous protoporphyrinogen IX oxidase (PPO) proteins: Evidence that small N-terminal amino acid changes do not impact protein function
Source: PLoS One. 2024 Sep 26;19(9):e0311049. doi: 10.1371/journal.pone.0311049 (PMC11426539; doi:10.1371/journal.pone.0311049)
Supplement: S2 File — Supplementary data include molecular weight determination and purity analysis for Table 2. The SDS-PAGE gel was analyzed by a Bio-Rad GS-900 Calibrated Densitometer using Bio-Rad Image Lab Security Edition Software version 6.1.0 build 7. (PDF) [file pone.0311049.s008.pdf]

Image Report: Table 2 Purity and Molecular Weight Analysis of Figure 2

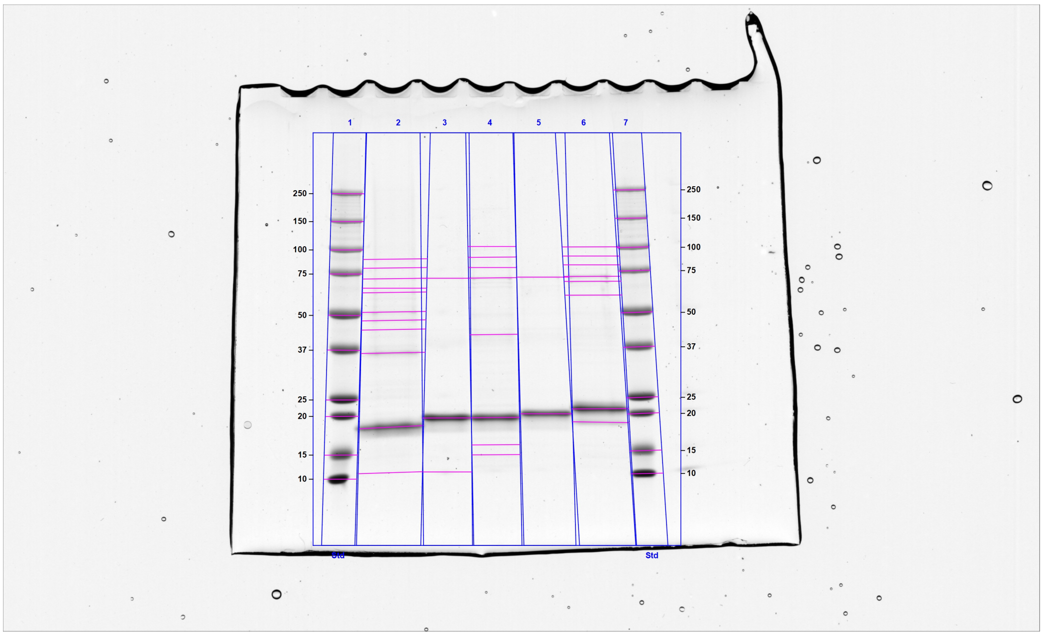

C:\Users\GOQRT\OneDrive - Bayer\Projects\2024\HT5 Soy\PPO\Manuscript\SDS-PAGE\Table 2 Purity and Molecular Weight Analysis of Figure 2.scn

---

Acquisition Information

Imager	GS-900
Serial Number	SD7WBA00047
Firmware Version	61
Software Version	6.1.0.07
Application	Coomassie Brilliant Blue R-250
Scan Mode	Transmissive
Scan Color	Red
OD Calibration	Yes
Flat Field	Red

Image Information

Acquisition Date	2/8/2024 3:22:05 PM
User Name	Chandler Est
Image Area (mm)	X: 172.0 Y: 104.0
Pixel Size (µm)	X: 63.5 Y: 63.5
Data Range (OD)	0.000 - 2.475

Analysis Settings

Detection	Lane detection:<br>Manually created lanes<br><br>Band detection:

	Manually adjusted bands<br><br>Lane Background Subtraction:<br>Lane background subtracted with disk size: 10<br><br>Lane width: Variable
Mol. Weight Analysis	Standard: Bio-Rad Precision Plus<br>Standard lanes: first last<br>Regression method: Point to Point (semi-log)

Calibration Report

OD Value	Mean Intensity	Max Value	Min Value	Std Dev
0.00	6584	57734	3665	691.80
0.07	16140	34366	14454	523.53
0.27	31843	38657	29967	423.47
0.46	43275	47855	41971	303.91
0.67	50824	54277	48453	228.43
0.87	55504	57254	54882	164.84
1.08	58914	59924	58355	115.65
1.27	61080	61797	60728	86.53
1.48	62548	62940	62252	66.64
1.69	63488	63710	63225	53.54
1.90	64121	64334	63941	43.12
2.11	64518	64674	64349	36.64
2.31	64771	64948	64615	32.05
2.51	64938	65099	64778	28.73
2.70	65060	65244	64924	26.93
2.90	65146	65336	64991	25.82
3.11	65201	65362	65053	25.18
3.32	65233	65438	65081	24.73
3.54	65254	65426	65067	24.79
3.72	65260	65442	65100	26.36

Note: Values reported are specular OD values to account for differences in how scanner-based instruments measure diffuse and specular samples.

Lane Statistics

Lane No.	Adj. Total Band Vol. (OD)	Total Band Vol. (OD)	Adj. Total Lane Vol. (OD)	Total Lane Vol. (OD)	Bkgd. Vol. (OD)	Norm. Factor
1	5,847.77	6,211.13	5,980.70	6,823.00	842.30	N/A
2	1,614.28	1,682.46	1,779.85	2,013.27	233.41	N/A
3	1,365.99	1,367.43	1,431.27	1,444.64	13.37	N/A
4	1,248.05	1,269.72	1,364.33	1,435.17	70.84	N/A
5	995.10	996.46	1,094.74	1,113.14	18.39	N/A
6	1,675.84	1,683.25	1,907.25	1,957.93	50.68	N/A
7	5,050.26	5,273.46	5,248.10	5,820.92	572.83	N/A

Lane And Band Analysis

Lane 1 - Bio-Rad Precision Plus

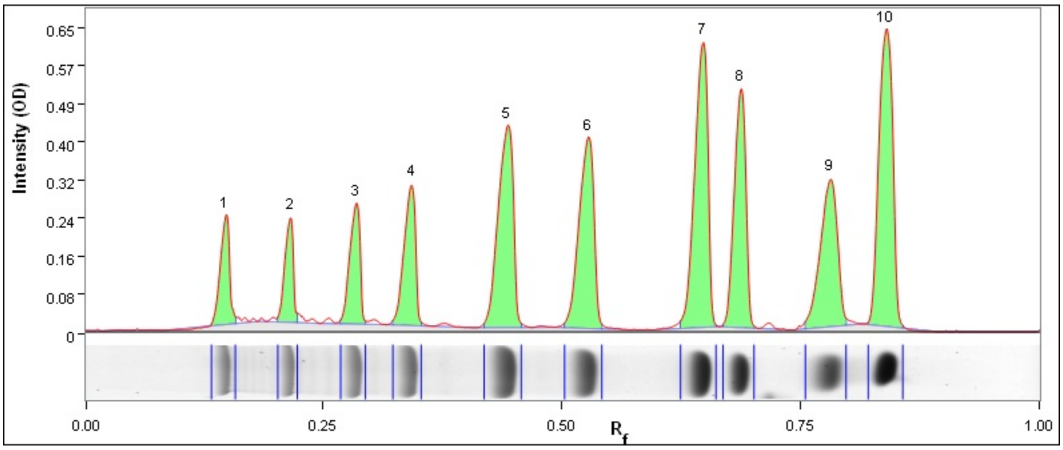

Band No.	Band Label	Mol. Wt. (KDa)	Relative Front	Adj. Volume (OD)	Volume (OD)	Abs. Quant.	Rel. Quant.	Band %	Lane %
1		250.0	0.147	248.25	285.01	N/A	N/A	4.2	4.2
2		150.0	0.215	217.37	258.22	N/A	N/A	3.7	3.6
3		100.0	0.284	306.98	348.98	N/A	N/A	5.2	5.1
4		75.0	0.342	393.09	432.77	N/A	N/A	6.7	6.6
5		50.0	0.442	751.58	785.40	N/A	N/A	12.9	12.6
6		37.0	0.527	696.14	723.61	N/A	N/A	11.9	11.6
7		25.0	0.647	924.64	956.54	N/A	N/A	15.8	15.5
8		20.0	0.687	679.02	707.60	N/A	N/A	11.6	11.4
9		15.0	0.781	620.08	661.45	N/A	N/A	10.6	10.4
10		10.0	0.840	1,010.61	1,051.56	N/A	N/A	17.3	16.9

Lane Background	Lane background subtracted with disk size: 10
Lane Width	5.52 mm
Regression Equation	A single equation is not available for this method

Lane 2

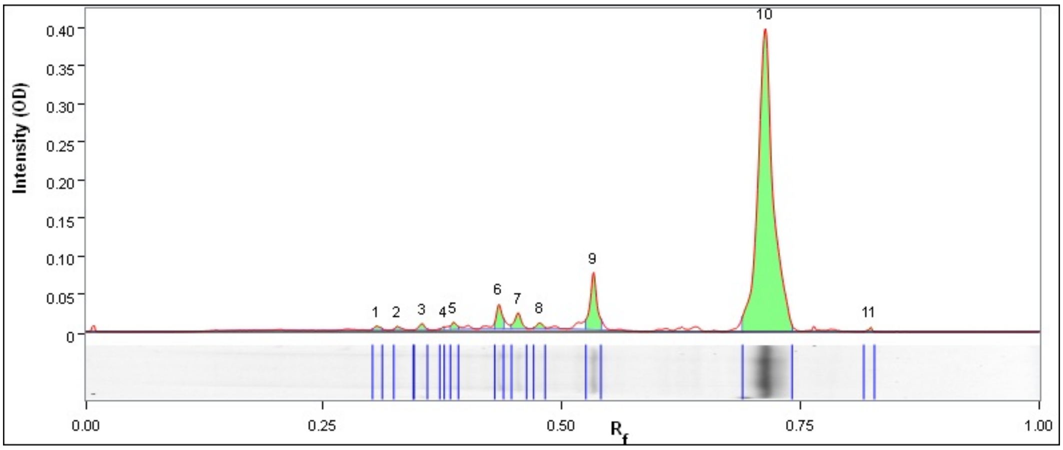

Band No.	Band Label	Mol. Wt. (KDa)	Relative Front	Adj. Volume (OD)	Volume (OD)	Abs. Quant.	Rel. Quant.	Band %	Lane %
1		89.1	0.306	7.86	11.95	N/A	N/A	0.5	0.4
2		80.1	0.327	6.98	15.48	N/A	N/A	0.4	0.4
3		71.3	0.353	11.37	17.27	N/A	N/A	0.7	0.6
4		64.9	0.377	3.22	5.74	N/A	N/A	0.2	0.2
5		62.3	0.387	11.64	16.54	N/A	N/A	0.7	0.7

6		51.4	0.434	39.96	47.01	N/A	N/A	2.5	2.2
7		47.7	0.455	31.45	43.31	N/A	N/A	1.9	1.8
8		44.0	0.477	11.39	20.63	N/A	N/A	0.7	0.6
9		36.1	0.533	111.02	118.64	N/A	N/A	6.9	6.2
10		18.4	0.713	1,374.75	1,380.80	N/A	N/A	85.2	77.2
11		11.0	0.824	4.64	5.07	N/A	N/A	0.3	0.3

Lane Background	Lane background subtracted with disk size: 10
Lane Width	10.67 mm
Regression Equation	A single equation is not available for this method

Lane 3

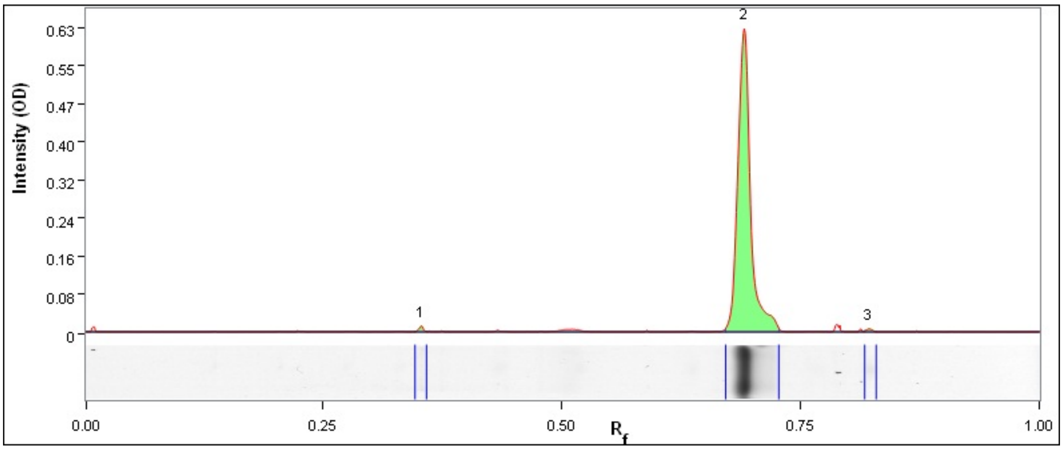

Band No.	Band Label	Mol. Wt. (KDa)	Relative Front	Adj. Volume (OD)	Volume (OD)	Abs. Quant.	Rel. Quant.	Band %	Lane %
1		71.1	0.353	8.59	9.10	N/A	N/A	0.6	0.6
2		19.6	0.691	1,351.26	1,352.14	N/A	N/A	98.9	94.4
3		10.9	0.822	6.14	6.20	N/A	N/A	0.4	0.4

Lane Background	Lane background subtracted with disk size: 10
Lane Width	8.13 mm
Regression Equation	A single equation is not available for this method

Lane 4

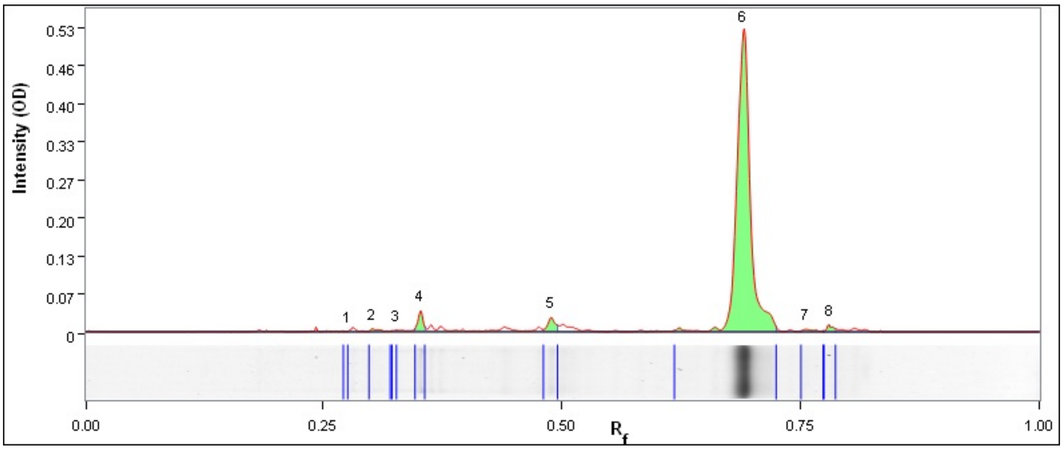

Band No.	Band Label	Mol. Wt. (KDa)	Relative Front	Adj. Volume (OD)	Volume (OD)	Abs. Quant.	Rel. Quant.	Band %	Lane %
1		103.0	0.276	0.21	0.78	N/A	N/A	0.0	0.0
2		90.1	0.301	5.58	8.68	N/A	N/A	0.4	0.4
3		79.5	0.327	0.92	1.85	N/A	N/A	0.1	0.1
4		71.0	0.352	28.80	30.90	N/A	N/A	2.3	2.1
5		41.8	0.489	28.54	30.41	N/A	N/A	2.3	2.1
6		19.6	0.690	1,164.90	1,176.00	N/A	N/A	93.3	85.4
7		15.9	0.756	7.36	8.78	N/A	N/A	0.6	0.5
8		14.5	0.780	11.74	12.31	N/A	N/A	0.9	0.9

Lane Background	Lane background subtracted with disk size: 10
Lane Width	8.00 mm
Regression Equation	A single equation is not available for this method

Lane 5

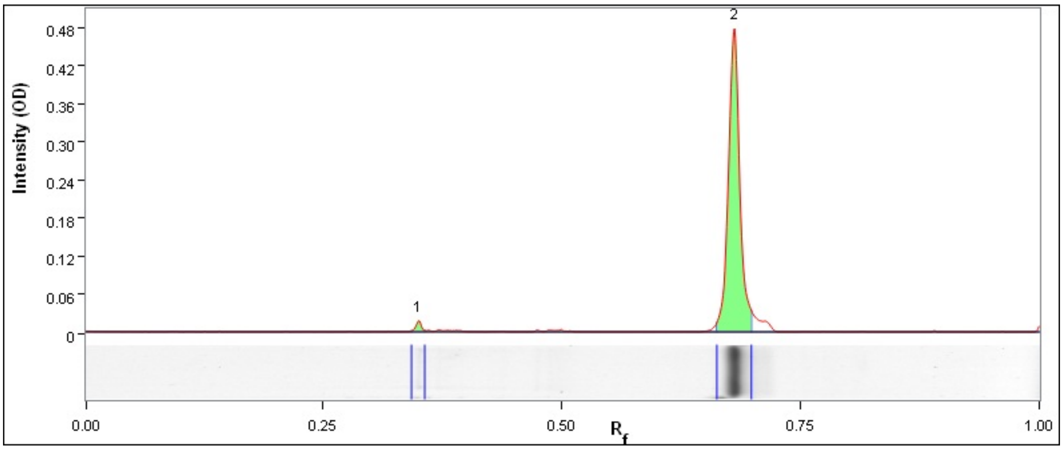

Band No.	Band Label	Mol. Wt. (KDa)	Relative Front	Adj. Volume (OD)	Volume (OD)	Abs. Quant.	Rel. Quant.	Band %	Lane %
1		71.1	0.350	16.20	17.36	N/A	N/A	1.6	1.5
2		20.2	0.681	978.90	979.10	N/A	N/A	98.4	89.4

Lane Background	Lane background subtracted with disk size: 10
Lane Width	8.64 mm
Regression Equation	A single equation is not available for this method

Lane 6

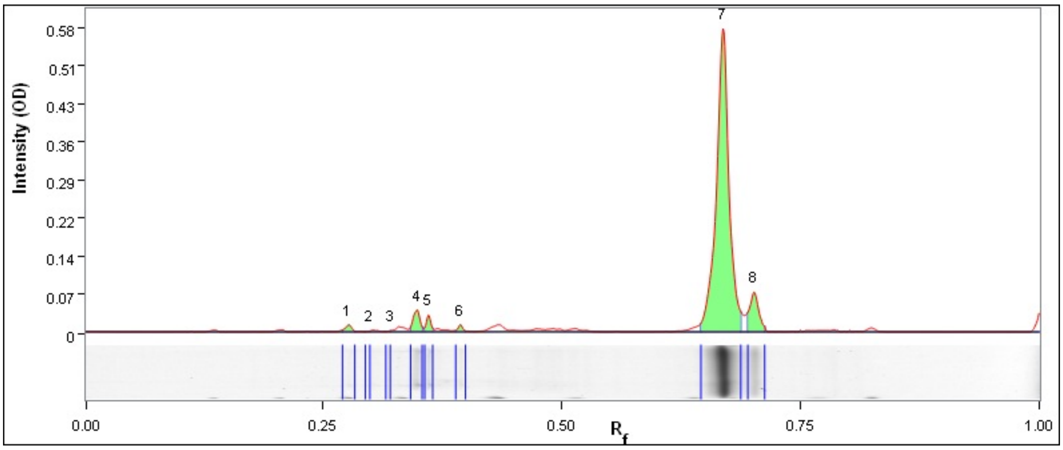

Band No.	Band Label	Mol. Wt. (KDa)	Relative Front	Adj. Volume (OD)	Volume (OD)	Abs. Quant.	Rel. Quant.	Band %	Lane %
1		101.1	0.276	15.78	16.78	N/A	N/A	0.9	0.8
2		90.2	0.299	0.55	1.01	N/A	N/A	0.0	0.0
3		81.0	0.320	0.79	1.41	N/A	N/A	0.0	0.0
4		71.3	0.348	53.61	55.06	N/A	N/A	3.2	2.8
5		67.9	0.360	26.70	27.73	N/A	N/A	1.6	1.4
6		59.4	0.393	11.19	12.43	N/A	N/A	0.7	0.6
7		21.4	0.669	1,434.84	1,435.94	N/A	N/A	85.6	75.2
8		18.8	0.701	132.39	132.89	N/A	N/A	7.9	6.9

Lane Background	Lane background subtracted with disk size: 10
Lane Width	9.46 mm
Regression Equation	A single equation is not available for this method

Lane 7 - Bio-Rad Precision Plus

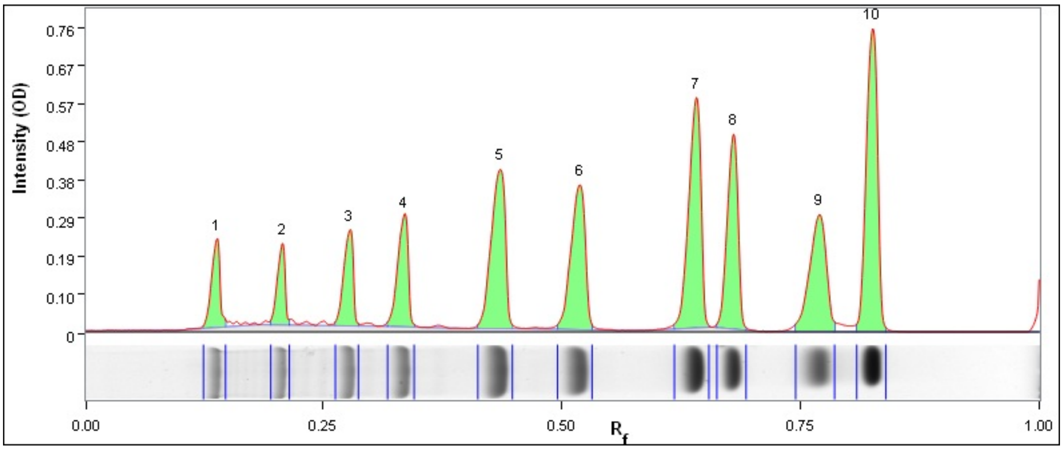

Band No.	Band Label	Mol. Wt. (KDa)	Relative Front	Adj. Volume (OD)	Volume (OD)	Abs. Quant.	Rel. Quant.	Band %	Lane %
1		250.0	0.138	210.96	233.63	N/A	N/A	4.2	4.0
2		150.0	0.207	177.61	209.43	N/A	N/A	3.5	3.4
3		100.0	0.277	255.43	289.67	N/A	N/A	5.1	4.9
4		75.0	0.334	329.98	363.09	N/A	N/A	6.5	6.3
5		50.0	0.435	627.33	655.48	N/A	N/A	12.4	12.0
6		37.0	0.519	575.45	593.61	N/A	N/A	11.4	11.0
7		25.0	0.641	784.31	814.99	N/A	N/A	15.5	14.9
8		20.0	0.680	585.79	606.94	N/A	N/A	11.6	11.2
9		15.0	0.770	543.54	545.89	N/A	N/A	10.8	10.4
10		10.0	0.826	959.86	960.74	N/A	N/A	19.0	18.3

Lane Background	Lane background subtracted with disk size: 10
Lane Width	5.33 mm
Regression Equation	A single equation is not available for this method
