# Supplementary material for: Production and characterization of homologous protoporphyrinogen IX oxidase (PPO) proteins: Evidence that small N-terminal amino acid changes do not impact protein function
Source: PLoS One. 2024 Sep 26;19(9):e0311049. doi: 10.1371/journal.pone.0311049 (PMC11426539; doi:10.1371/journal.pone.0311049)
Supplement: S3 File — Supplementary data for Table 2 include tandem mass spectrometry (MS/MS) spectra of the N-terminal and C-terminal peptides, as well as sequence coverage for E. coli-produced PPO variants. (PDF) [file pone.0311049.s009.pdf]

Tag-free PPO Coverage

MASCOT Search Results

Protein View: Tag-free PPO

Tag-free PPO

**Database:**	Tag-freePPO
**Score:**	22498
**Monoisotopic mass (M_r):**	20809
**Calculated pI:**	9.11

Sequence similarity is available as **an NCBI BLAST search of Tag-free PPO against nr**.

Search parameters

**MS data file:**	C:\Users\EKYSC\Desktop\20231207_Tag-freePPO.raw
**Enzyme:**	Chymotrypsin: cuts C-term side of FLWY unless next residue is P.
**Fixed modifications:**	**Carbamidomethyl (C)**
**Variable modifications:**	**Oxidation (M)**

Protein sequence coverage: 100%

Matched peptides shown in ***bold red***.

```
  1 MKALVLYSTR DGQTHAIASY IASCMKEKAE CDVIDLTHGE HVNLTQYDQV
 51 LIGASIRYGH FNAVLDKFIK RNVDQLNNMP SAFFCVNLTA RKPEKRTPQT
101 NPYVRKFLLA TPWQPALCGV FAGALRYPRY RWIDKVMIQL IMRMTGGETD
151 TSKEVEYTDW EQVKKFAEDF AKLSYKKAL
```

Tag-free PPO N-term

Mascot Search Results

Peptide View

MS/MS Fragmentation of **MKALVLY**
Found in **Tag-free PPO** in **Tag-freePPO**, Tag-free PPO

Match to Query 8784: 836.482342 from(419.248447,2+) intensity(8940942144.1250) rtinseconds(2147.885178) index(20375)
Title: 20231207_Tag-freePPO.22256.22256.2
Data file C:\Users\EKYSC\Desktop\20231207_Tag-freePPO.raw

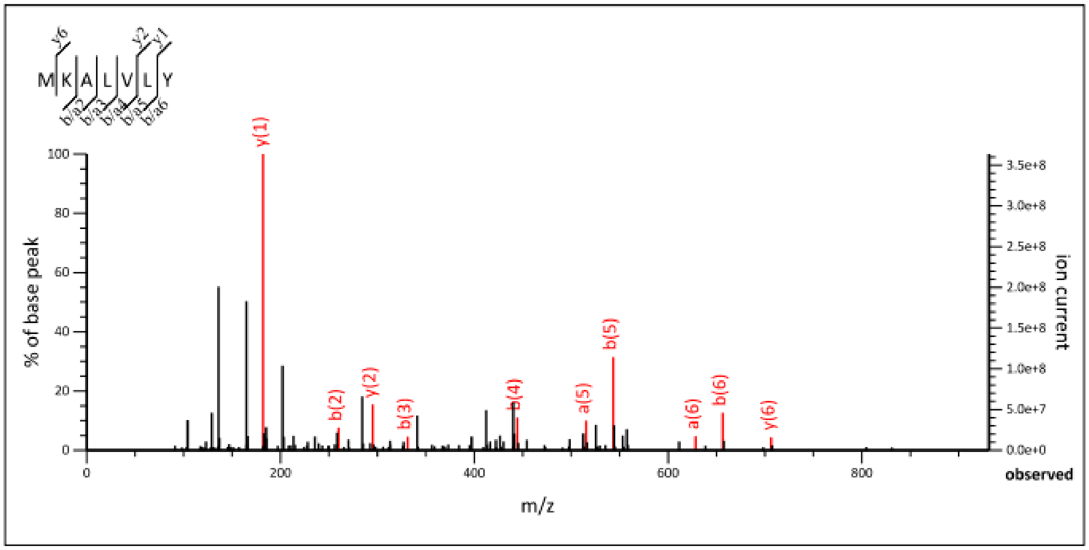

Label all possible matches ○ Label matches used for scoring ◉

```
Monoisotopic mass of neutral peptide Mr(calc): 836.4830
Fixed modifications: Carbamidomethyl (C) (apply to specified residues or termini only)
Ions Score: 25  Expect: 0.0032
Matches : 10/58 fragment ions using 23 most intense peaks   (help)
```

#	a	a++	a*	a*++	b	b++	b*	b*++	Seq.	y	y++	y*	y*++	#
1	104.0528	52.5301			132.0478	66.5275			M					7
2	232.1478	116.5775	215.1213	108.0643	***260.1427***	130.5750	243.1162	122.0617	K	**706.4498**	353.7285	689.4232	345.2153	6
3	303.1849	152.0961	286.1584	143.5828	***331.1798***	166.0936	314.1533	157.5803	A	578.3548	289.6811			5
4	416.2690	208.6381	399.2424	200.1249	***444.2639***	222.6356	427.2374	214.1223	L	507.3177	254.1625			4
5	**515.3374**	258.1723	498.3109	249.6591	***543.3323***	272.1698	526.3058	263.6565	V	394.2336	197.6205			3
6	**628.4215**	314.7144	611.3949	306.2011	***656.4164***	328.7118	639.3898	320.1986	L	**295.1652**	148.0863			2
7									Y	**182.0812**	91.5442			1

Tag-free PPO C-term

Mascot Search Results

Peptide View

MS/MS Fragmentation of **AEDFAKLSYKKAL**
Found in **Tag-free PPO** in **Tag-freePPO**, Tag-free PPO

Match to Query 23634: 1482.808629 from(495.276819,3+) intensity(19026546.6250) rtinseconds(1735.886529) index(16143)
Title: 20231207_Tag-freePPO.17759.17759.3
Data file C:\Users\EKYSC\Desktop\20231207_Tag-freePPO.raw

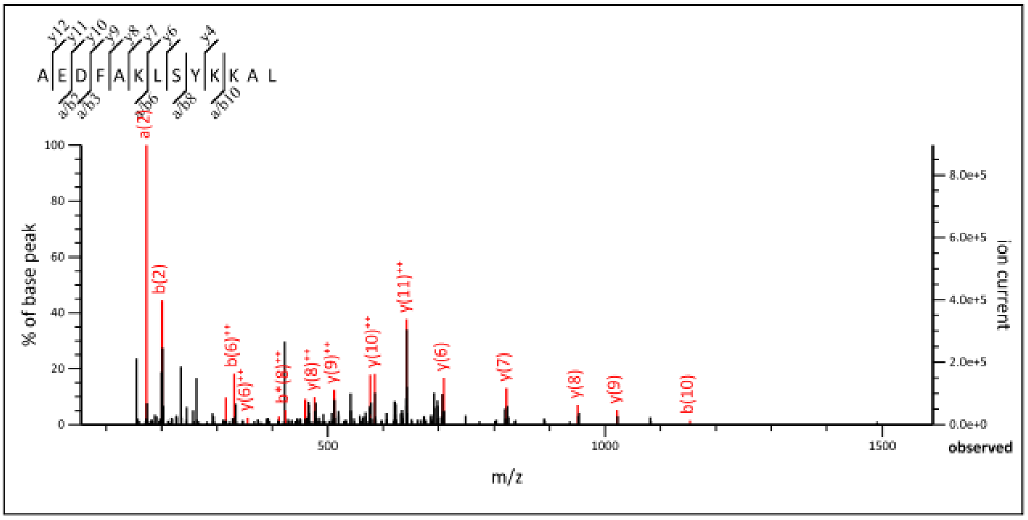

Label all possible matches ○ Label matches used for scoring ◉

```
Monoisotopic mass of neutral peptide Mr(calc): 1482.8082
Fixed modifications: Carbamidomethyl (C) (apply to specified residues or termini only)
Ions Score: 39  Expect: 0.00012
Matches : 21/120 fragment ions using 37 most intense peaks   (help)
```

#	a	a^{++}	a*	a*$^{++}$	b	b^{++}	b*	b*$^{++}$	Seq.	y	y^{++}	y*	y*$^{++}$	#
1	44.0495	22.5284			72.0444	36.5258			A					13
2	173.0921	87.0497			**201.0870**	101.0471			E	1412.7784	***706.8928***	1395.7518	698.3796	12
3	288.1190	144.5631			**316.1139**	158.5606			D	1283.7358	***642.3715***	1266.7093	633.8583	11
4	435.1874	218.0974			463.1823	232.0948			F	1168.7089	***584.8581***	1151.6823	576.3448	10
5	506.2245	253.6159			534.2195	267.6134			A	***1021.6404***	***511.3239***	1004.6139	502.8106	9
6	634.3195	317.6634	617.2930	309.1501	662.3144	**331.6608**	645.2879	323.1476	K	***950.6033***	***475.8053***	933.5768	467.2920	8
7	747.4036	374.2054	730.3770	365.6921	775.3985	388.2029	758.3719	379.6896	L	***822.5084***	***411.7578***	805.4818	403.2445	7
8	834.4356	417.7214	817.4090	409.2082	862.4305	431.7189	845.4040	423.2056	S	***709.4243***	***355.2158***	692.3978	346.7025	6
9	997.4989	499.2531	980.4724	490.7398	1025.4938	513.2506	1008.4673	504.7373	Y	622.3923	311.6998	605.3657	303.1865	5
10	1125.5939	563.3006	1108.5673	554.7873	**1153.5888**	**577.2980**	1136.5623	568.7848	K	***459.3289***	230.1681	442.3024	221.6548	4
11	1253.6888	627.3481	1236.6623	618.8348	1281.6838	641.3455	1264.6572	632.8322	K	***331.2340***	166.1206	314.2074	157.6074	3

PPO Coverage

MASCOT Search Results

Protein View: PPO

PPO

**Database:**	PPO
**Score:**	19109
**Monoisotopic mass (M_r):**	21631
**Calculated pI:**	9.11

Sequence similarity is available as **an NCBI BLAST search of PPO against nr**.

Search parameters

**MS data file:**	C:\Users\EKYSC\Desktop\20231207_PPO.raw
**Enzyme:**	Chymotrypsin: cuts C-term side of FLWY unless next residue is P.
**Fixed modifications:**	**Carbamidomethyl (C)**
**Variable modifications:**	**Oxidation (M)**

Protein sequence coverage: 100%

Matched peptides shown in ***bold red***.

```
  1 MHHHHHHKAL VLYSTRDGQT HAIASYIASC MKEKAECDVI DLTHGEHVNL
 51 TQYDQVLIGA SIRYGHFNAV LDKFIKRNVD QLNNMPSAFF CVNLTARKPE
101 KRTPQTNPYV RKFLLATPWQ PALCGVFAGA LRYPRYRWID KVMIQLIMRM
151 TGGETDTSKE VEYTDWEQVK KFAEDFAKLS YKKAL
```

PPO N-term

Mascot Search Results

Peptide View

MS/MS Fragmentation of **MHHHHHHKALVLY**
Found in **PPO** in **PPO**, PPO

Match to Query 32294: 1674.832352 from(419.715364,4+) intensity(2333080.7891) rtinseconds(1872.307438) index(16834)
Title: 20231207_PPO.18735.18735.4
Data file C:\Users\EKYSC\Desktop\20231207_PPO.raw

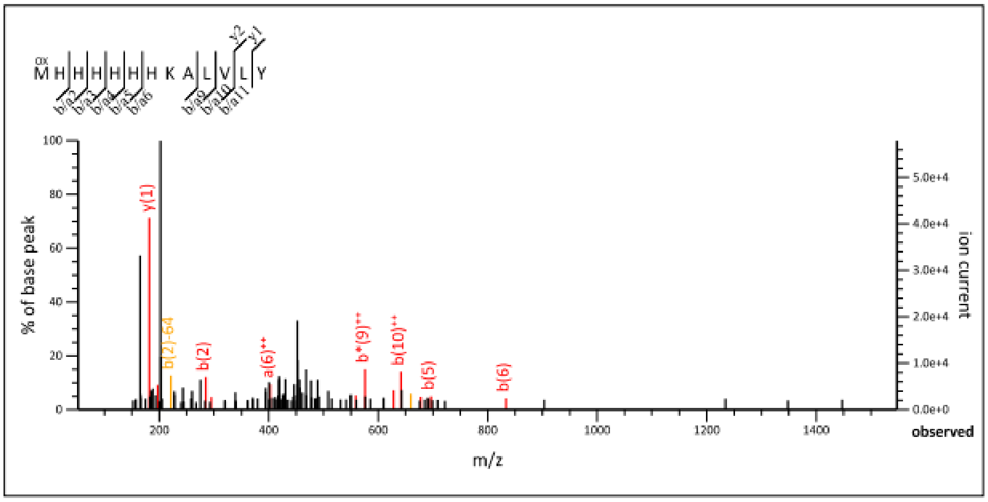

Label all possible matches ○ Label matches used for scoring ◉

Monoisotopic mass of neutral peptide Mr(calc): 1674.8314
Fixed modifications: Carbamidomethyl (C) (apply to specified residues or termini only)
Variable modifications:
M1 : Oxidation (M), with neutral losses 0.0000(shown in table), 63.9983
Ions Score: 13 **Expect:** 0.047
Matches : 19/174 fragment ions using 41 most intense peaks (help)

#	a	a++	a*	a*++	b	b++	b*	b*++	Seq.	y	y++	y*	y*++	#
1	120.0478	60.5275			148.0427	74.5250			M					13
2	257.1067	129.0570			***285.1016***	143.0544			H	1528.8033	764.9053	1511.7767	756.3920	12
3	394.1656	***197.5864***			422.1605	211.5839			H	1391.7443	**696.3758**	1374.7178	687.8625	11
4	531.2245	266.1159			***559.2194***	280.1133			H	1254.6854	**627.8464**	1237.6589	619.3331	10
5	668.2834	334.6453			***696.2783***	348.6428			H	1117.6265	**559.3169**	1100.6000	550.8036	9
6	805.3423	***403.1748***			***833.3372***	417.1723			H	980.5676	490.7874	963.5411	482.2742	8
7	942.4012	471.7043			970.3961	485.7017			H	843.5087	422.2580	826.4822	413.7447	7
8	1070.4962	535.7517	1053.4696	527.2385	1098.4911	549.7492	1081.4646	541.2359	K	706.4498	353.7285	689.4232	345.2153	6
9	1141.5333	571.2703	1124.5068	562.7570	1169.5282	585.2678	1152.5017	576.7545	A	578.3548	289.6811			5
10	1254.6174	***627.8123***	1237.5908	619.2991	1282.6123	**641.8098**	1265.5857	633.2965	L	507.3177	254.1625			4
11	1353.6858	***677.3465***	1336.6592	668.8333	1381.6807	**691.3440**	1364.6542	682.8307	V	394.2336	**197.6205**			3
12	1466.7699	733.8886	1449.7433	725.3753	1494.7648	747.8860	1477.7382	739.3727	L	**295.1652**	148.0863			2
13									Y	**182.0812**	91.5442			1

PPO C-term

Mascot Search Results

Peptide View

MS/MS Fragmentation of **AEDFAKLSYKKAL**
Found in **PPO** in **PPO**, PPO

Match to Query 27330: 1482.807970 from(742.411261,2+) intensity(3355174.8750) rtinseconds(1741.577343) index(15493)
Title: 20231207_PPO.17310.17310.2
Data file C:\Users\EKYSC\Desktop\20231207_PPO.raw

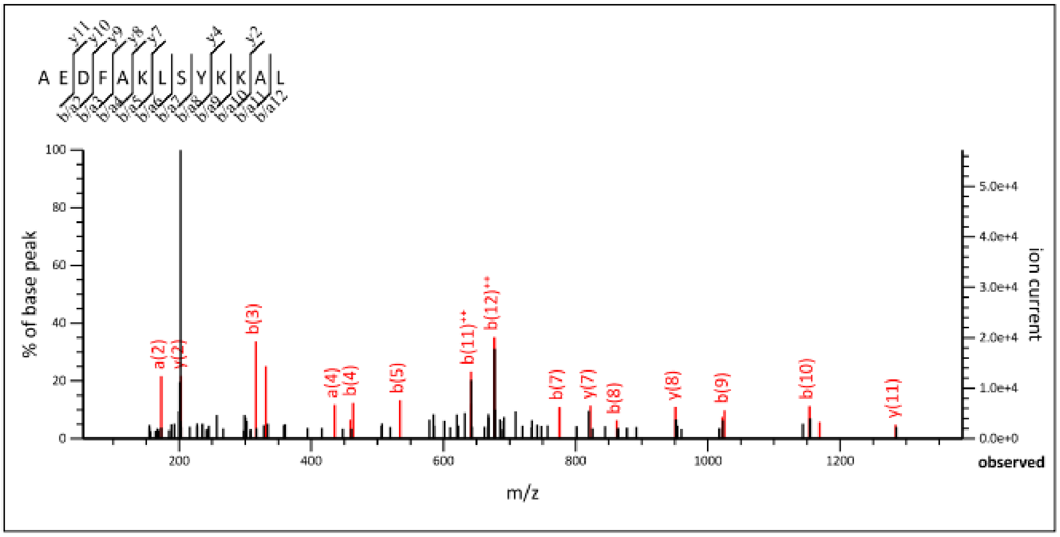

Label all possible matches ○ Label matches used for scoring ◉

Monoisotopic mass of neutral peptide Mr(calc): 1482.8082
Fixed modifications: Carbamidomethyl (C) (apply to specified residues or termini only)
Ions Score: 53 **Expect:** 4.9e-06
Matches : 21/120 fragment ions using 33 most intense peaks (help)

#	a	a^{++}	a*	a^{*++}	b	b^{++}	b*	b^{*++}	Seq.	y	y^{++}	y*	y^{*++}	#
1	44.0495	22.5284			72.0444	36.5258			A					13
2	**173.0921**	87.0497			201.0870	101.0471			E	1412.7784	706.8928	1395.7518	698.3796	12
3	288.1190	144.5631			**316.1139**	158.5606			D	**1283.7358**	642.3715	1266.7093	633.8583	11
4	**435.1874**	218.0974			**463.1823**	232.0948			F	**1168.7089**	584.8581	1151.6823	576.3448	10
5	506.2245	253.6159			**534.2195**	267.6134			A	**1021.6404**	511.3239	1004.6139	502.8106	9
6	634.3195	317.6634	617.2930	309.1501	662.3144	**331.6608**	645.2879	323.1476	K	**950.6033**	475.8053	933.5768	467.2920	8
7	747.4036	374.2054	730.3770	365.6921	**775.3985**	388.2029	758.3719	379.6896	L	**822.5084**	411.7578	805.4818	403.2445	7
8	834.4356	417.7214	817.4090	409.2082	**862.4305**	431.7189	845.4040	423.2056	S	709.4243	355.2158	692.3978	346.7025	6
9	997.4989	499.2531	980.4724	490.7398	**1025.4938**	513.2506	1008.4673	504.7373	Y	622.3923	311.6998	605.3657	303.1865	5
10	1125.5939	563.3006	1108.5673	554.7873	**1153.5888**	577.2980	1136.5623	568.7848	K	**459.3289**	230.1681	442.3024	221.6548	4
11	1253.6888	627.3481	1236.6623	618.8348	1281.6838	**641.3455**	1264.6572	632.8322	K	**331.2340**	166.1206	314.2074	157.6074	3
12	1324.7260	662.8666	1307.6994	654.3533	1352.7209	**676.8641**	1335.6943	668.3508	A	**203.1390**	102.0731			2
13									L	132.1019	66.5546			1

mPPO Coverage

MASCOT Search Results

Protein View: mPPO

mPPO

**Database:**	mPPO
**Score:**	17681
**Monoisotopic mass (M_r):**	23324
**Calculated pI:**	9.36

Sequence similarity is available as **an NCBI BLAST search of mPPO against nr**.

Search parameters

**MS data file:**	C:\Users\EKYSC\Desktop\20231207_mPPO.raw
**Enzyme:**	Chymotrypsin: cuts C-term side of FLWY unless next residue is P.
**Fixed modifications:**	Carbamidomethyl (C)
**Variable modifications:**	Oxidation (M)

Protein sequence coverage: 97%

Matched peptides shown in ***bold red***.

```
  1 MHHHHHHHTRR LDHRPFVVRC KALVLYSTRD GQTHAIASYI ASCMKEKAEC
 51 DVIDLTHGEH VNLTQYDQVL IGASIRYGHF NAVLDKFIKR NVDQLNNMPS
101 AFFCVNLTAR KPEKRTPQTN PYVRKFLLAT PWQPALCGVF AGALRYPRYR
151 WIDKVMIQLI MRMTGGETDT SKEVEYTDWE QVKKFAEDFA KLSYKKAL
```

mPPO N-term

Mascot Search Results

Peptide View

MS/MS Fragmentation of **MHHHHHHTRRL**
Found in **mPPO** in **mPPO**, mPPO

Match to Query 28411: 1497.738456 from(375.441890,4+) intensity(3069549.7813) rtinseconds(1872.646376) index(17718)
Title: 20231207_mPPO.19354.19354.4
Data file C:\Users\EKYSC\Desktop\20231207_mPPO.raw

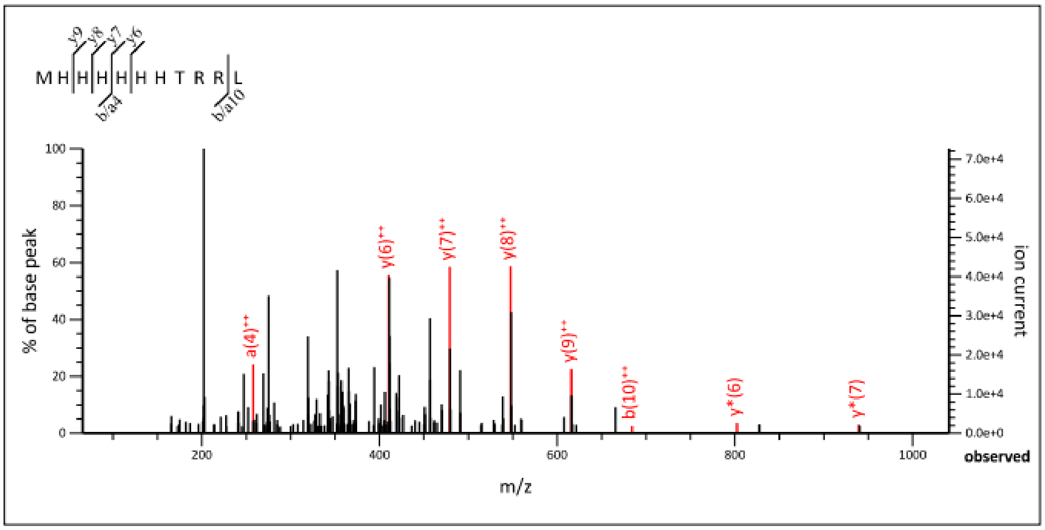

Label all possible matches ○ Label matches used for scoring ◉

Monoisotopic mass of neutral peptide Mr(calc): 1497.7385
Fixed modifications: Carbamidomethyl (C) (apply to specified residues or termini only)
Ions Score: 19 **Expect:** 0.014
Matches : 9/86 fragment ions using 15 most intense peaks (help)

#	a	a^{++}	a*	a^{*++}	b	b^{++}	b*	b^{*++}	Seq.	y	y^{++}	y*	y^{*++}	#
1	104.0528	52.5301			132.0478	66.5275			M					11
2	241.1118	121.0595			269.1067	135.0570			H	1367.7053	***684.3563***	1350.6787	675.8430	10
3	378.1707	189.5890			406.1656	203.5864			H	1230.6464	***615.8268***	1213.6198	607.3135	9
4	515.2296	258.1184			543.2245	272.1159			H	1093.5875	***547.2974***	1076.5609	538.7841	8
5	652.2885	326.6479			680.2834	340.6453			H	956.5285	***478.7679***	939.5020	470.2546	7
6	789.3474	395.1773			817.3423	409.1748			H	819.4696	***410.2385***	802.4431	401.7252	6
7	926.4063	463.7068			954.4012	477.7043			H	682.4107	341.7090	665.3842	333.1957	5
8	1027.4540	514.2306			1055.4489	528.2281			T	545.3518	273.1795	528.3253	264.6663	4
9	1183.5551	592.2812	1166.5286	583.7679	1211.5500	606.2786	1194.5235	597.7654	R	444.3041	222.6557	427.2776	214.1424	3
10	1339.6562	670.3317	1322.6297	661.8185	1367.6511	684.3292	1350.6246	675.8159	R	288.2030	144.6051	271.1765	136.0919	2
11									L	132.1019	66.5546			1

mPPO C-term

Mascot Search Results

Peptide View

MS/MS Fragmentation of **AEDFAKLSYKKAL**
Found in **mPPO** in **mPPO**, mPPO

Match to Query 28168: 1482.808436 from(742.411494,2+) intensity(5162765.6250) rtinseconds(1745.110351) index(16410)
Title: 20231207_mPPO.17964.17964.2
Data file C:\Users\EKYSC\Desktop\20231207_mPPO.raw

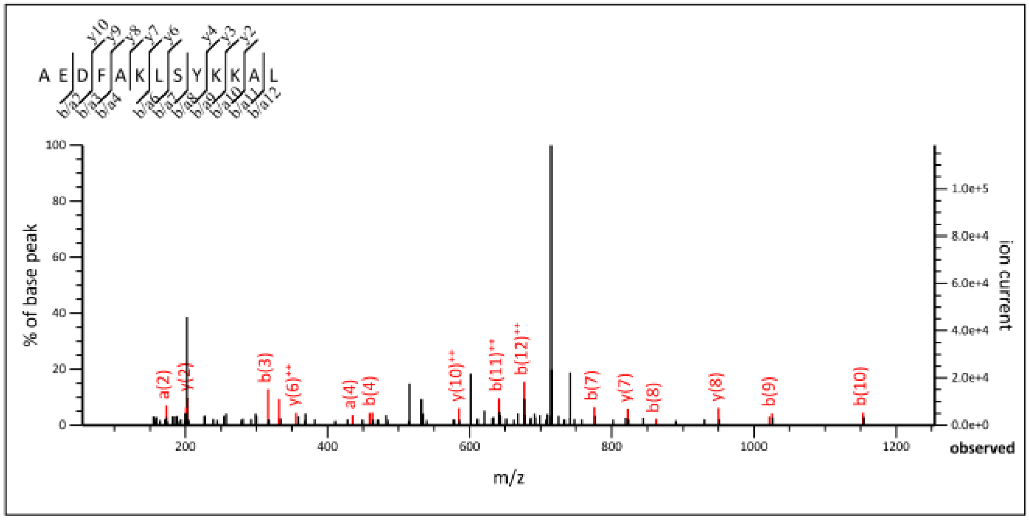

Label all possible matches ○ Label matches used for scoring ◉

Monoisotopic mass of neutral peptide Mr(calc): 1482.8082
Fixed modifications: Carbamidomethyl (C) (apply to specified residues or termini only)
Ions Score: 41 **Expect:** 8.2e-05
Matches : 20/120 fragment ions using 37 most intense peaks (help)

#	a	a++	a*	a*++	b	b++	b*	b*++	Seq.	y	y++	y*	y*++	#
1	44.0495	22.5284			72.0444	36.5258			A					13
2	**173.0921**	87.0497			***201.0870***	101.0471			E	1412.7784	706.8928	1395.7518	698.3796	12
3	288.1190	144.5631			***316.1139***	158.5606			D	1283.7358	642.3715	1266.7093	633.8583	11
4	**435.1874**	218.0974			***463.1823***	232.0948			F	1168.7089	**584.8581**	1151.6823	576.3448	10
5	506.2245	253.6159			534.2195	267.6134			A	***1021.6404***	511.3239	1004.6139	502.8106	9
6	634.3195	317.6634	617.2930	309.1501	662.3144	**331.6608**	645.2879	323.1476	K	***950.6033***	475.8053	933.5768	467.2920	8
7	747.4036	374.2054	730.3770	365.6921	***775.3985***	388.2029	758.3719	379.6896	L	***822.5084***	411.7578	805.4818	403.2445	7
8	834.4356	417.7214	817.4090	409.2082	***862.4305***	431.7189	845.4040	423.2056	S	709.4243	**355.2158**	692.3978	346.7025	6
9	997.4989	499.2531	980.4724	490.7398	***1025.4938***	513.2506	1008.4673	504.7373	Y	622.3923	311.6998	605.3657	303.1865	5
10	1125.5939	563.3006	1108.5673	554.7873	***1153.5888***	577.2980	1136.5623	568.7848	K	***459.3289***	230.1681	442.3024	221.6548	4
11	1253.6888	627.3481	1236.6623	618.8348	1281.6838	**641.3455**	1264.6572	632.8322	K	***331.2340***	166.1206	314.2074	157.6074	3
12	1324.7260	662.8666	1307.6994	654.3533	1352.7209	**676.8641**	1335.6943	668.3508	A	***203.1390***	102.0731			2
13									L	132.1019	66.5546			1

cPPO Sequence Coverage

MASCOT Search Results

Protein View: cPPO

cPPO

**Database:**	cPPO
**Score:**	21410
**Monoisotopic mass (M_r):**	21762
**Calculated pI:**	9.11

Sequence similarity is available as **an NCBI BLAST search of cPPO against nr**.

Search parameters

**MS data file:**	C:\Users\EKYSC\Desktop\20231207_cPPO.raw
**Enzyme:**	Chymotrypsin: cuts C-term side of FLWY unless next residue is P.
**Fixed modifications:**	Carbamidomethyl (C)
**Variable modifications:**	Oxidation (M)

Protein sequence coverage: 100%

Matched peptides shown in ***bold red***.

```
  1 MHHHHHHMKA LVLYSTRDGQ THAIASYIAS CMKEKAECDV IDLTHGEHVN
 51 LTQYDQVLIG ASIRYGHFNA VLDKFIKRNV DQLNNMPSAF FCVNLTARKP
101 EKRTPQTNPY VRKFLLATPW QPALCGVFAG ALRYPRYRWI DKVMIQLIMR
151 MTGGETDTSK EVEYTDWEQV KKFAEDFAKL SYKKAL
```

[cPPO N-terminal Mass Spectra]

Mascot Search Results

Peptide View

MS/MS Fragmentation of **MHHHHHHMKALVLY**
Found in **cPPO** in **cPPO**, cPPO

Match to Query 35082: 1805.871192 from(452.475074,4+) intensity(17535901.0000) rtinseconds(2193.40676) index(20131)
Title: 20231207_cPPO.22238.22238.4
Data file C:\Users\EKYSC\Desktop\20231207_cPPO.raw

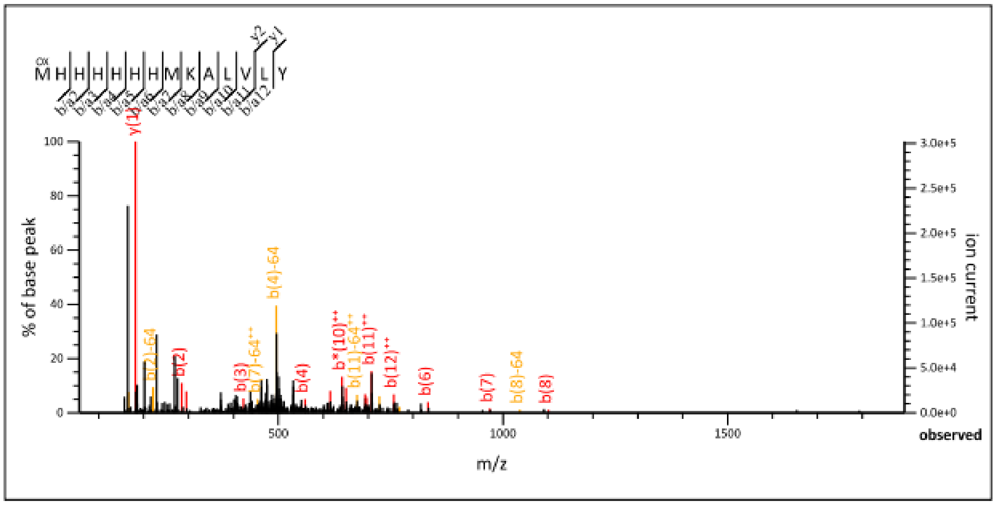

Label all possible matches ○ Label matches used for scoring ◉

Monoisotopic mass of neutral peptide Mr(calc): 1805.8719
Fixed modifications: Carbamidomethyl (C) (apply to specified residues or termini only)
Variable modifications:
M1 : Oxidation (M), with neutral losses 0.0000(shown in table), 63.9983
Ions Score: 22 **Expect:** 0.006
Matches : 24/186 fragment ions using 64 most intense peaks (help)

#	a	a++	a*	a*++	b	b++	b*	b*++	Seq.	y	y++	y*	y*++	#
1	120.0478	60.5275			148.0427	74.5250			M					14
2	257.1067	129.0570			***285.1016***	143.0544			H	1659.8437	830.4255	1642.8172	821.9122	13
3	394.1656	197.5864			***422.1605***	211.5839			H	1522.7848	761.8961	1505.7583	753.3828	12
4	531.2245	266.1159			***559.2194***	280.1133			H	1385.7259	693.3666	1368.6994	684.8533	11
5	668.2834	334.6453			***696.2783***	348.6428			H	1248.6670	624.8371	1231.6405	616.3239	10
6	805.3423	403.1748			***833.3372***	417.1723			H	1111.6081	556.3077	1094.5815	547.7944	9
7	942.4012	471.7043			***970.3961***	485.7017			H	974.5492	487.7782	957.5226	479.2650	8
8	1073.4417	537.2245			***1101.4366***	551.2220			M	837.4903	419.2488	820.4637	410.7355	7
9	1201.5367	601.2720	1184.5101	592.7587	1229.5316	***615.2694***	1212.5050	606.7562	K	706.4498	353.7285	689.4232	345.2153	6
10	1272.5738	636.7905	1255.5472	628.2773	1300.5687	***650.7880***	1283.5422	642.2747	A	578.3548	289.6811			5
11	1385.6579	693.3326	1368.6313	684.8193	1413.6528	***707.3300***	1396.6262	698.8168	L	507.3177	254.1625			4
12	1484.7263	742.8668	1467.6997	734.3535	1512.7212	***756.8642***	1495.6946	748.3510	V	394.2336	197.6205			3
13	1597.8103	799.4088	1580.7838	790.8955	1625.8053	813.4063	1608.7787	804.8930	L	295.1652	148.0863			2
14									Y	182.0812	91.5442			1

cPPO C-terminal Mass Spectra

Mascot Search Results

Peptide View

MS/MS Fragmentation of **AEDFAKLSYKKAL**
Found in **cPPO** in **cPPO**, cPPO

Match to Query 27332: 1482.808012 from(371.709279,4+) intensity(51001978.3438) rtinseconds(1743.910387) index(15515)
Title: 20231207_cPPO.17334.17334.4
Data file C:\Users\EKYSC\Desktop\20231207_cPPO.raw

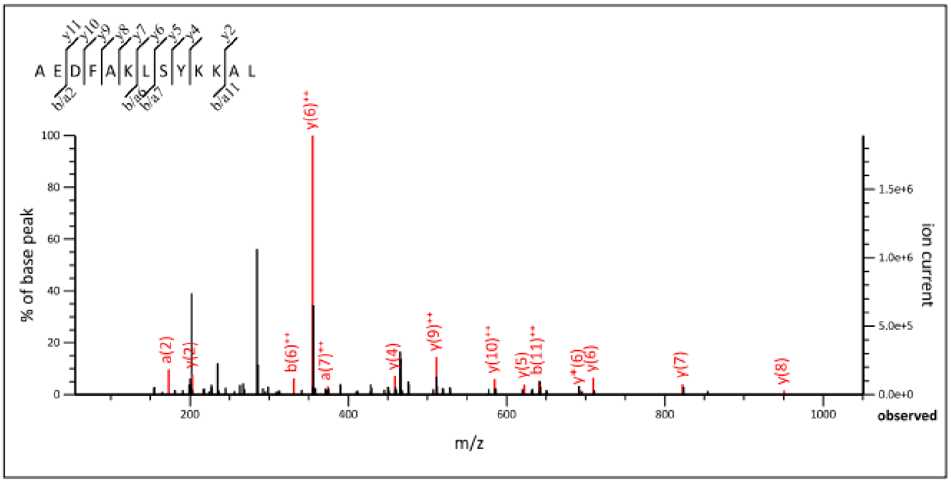

Label all possible matches ○ Label matches used for scoring ◉

Monoisotopic mass of neutral peptide Mr(calc): 1482.8082
Fixed modifications: Carbamidomethyl (C) (apply to specified residues or termini only)
Ions Score: 32 **Expect:** 0.00057
Matches : 16/120 fragment ions using 34 most intense peaks (help)

#	a	a++	a*	a*++	b	b++	b*	b*++	Seq.	y	y++	y*	y*++	#
1	44.0495	22.5284			72.0444	36.5258			A					13
2	173.0921	87.0497			201.0870	101.0471			E	1412.7784	706.8928	1395.7518	698.3796	12
3	288.1190	144.5631			316.1139	158.5606			D	1283.7358	*642.3715*	1266.7093	633.8583	11
4	435.1874	218.0974			463.1823	232.0948			F	1168.7089	*584.8581*	1151.6823	576.3448	10
5	506.2245	253.6159			534.2195	267.6134			A	1021.6404	*511.3239*	1004.6139	502.8106	9
6	634.3195	317.6634	617.2930	309.1501	662.3144	**331.6608**	645.2879	323.1476	K	*950.6033*	475.8053	933.5768	467.2920	8
7	747.4036	374.2054	730.3770	365.6921	775.3985	388.2029	758.3719	379.6896	L	*822.5084*	411.7578	805.4818	403.2445	7
8	834.4356	417.7214	817.4090	409.2082	862.4305	431.7189	845.4040	423.2056	S	*709.4243*	*355.2158*	692.3978	346.7025	6
9	997.4989	499.2531	980.4724	490.7398	1025.4938	513.2506	1008.4673	504.7373	Y	*622.3923*	311.6998	605.3657	303.1865	5
10	1125.5939	563.3006	1108.5673	554.7873	1153.5888	577.2980	1136.5623	568.7848	K	*459.3289*	230.1681	442.3024	221.6548	4
11	1253.6888	627.3481	1236.6623	618.8348	1281.6838	**641.3455**	1264.6572	632.8322	K	*331.2340*	166.1206	314.2074	157.6074	3
12	1324.7260	662.8666	1307.6994	654.3533	1352.7209	676.8641	1335.6943	668.3508	A	*203.1390*	102.0731			2
13									L	132.1019	66.5546			1

sPPO Sequence Coverage

MASCOT Search Results

Protein View: sPPO

sPPO

**Database:**	sPPO
**Score:**	24367
**Monoisotopic mass (M_r):**	21904
**Calculated pI:**	8.98

Sequence similarity is available as an NCBI BLAST search of sPPO against nr.

Search parameters

**MS data file:**	C:\Users\EKYSC\Desktop\20231207_sPPO.raw
**Enzyme:**	Chymotrypsin: cuts C-term side of FLWY unless next residue is P.
**Fixed modifications:**	Carbamidomethyl (C)
**Variable modifications:**	Oxidation (M)

Protein sequence coverage: 100%

Matched peptides shown in ***bold red***.

```
  1 MHHHHHHDAS KALVLYSTRD GQTHAIASYI ASCMKEKAEC DVIDLTHGEH
 51 VNLTQYDQVL IGASIRYGHF NAVLDKFIKR NVDQLNNMPS AFFCVNLTAR
101 KPEKRTPQTN PYVRKFLLAT PWQPALCGVF AGALRYPRYR WIDKVMIQLI
151 MRMTGGETDT SKEVEYTDWE QVKKFAEDFA KLSYKKAL
```

sPPO N-terminal Mass Spectra

Mascot Search Results

Peptide View

MS/MS Fragmentation of **MHHHHHHDASKALVLY**
Found in **sPPO** in **sPPO**, sPPO

Match to Query 35892: 1947.925764 from(487.988717,4+) intensity(1833371.6875) rtinseconds(3664.869792) index(35097)
Title: 20231207_sPPO.38187.38187.4
Data file C:\Users\EKYSC\Desktop\20231207_sPPO.raw

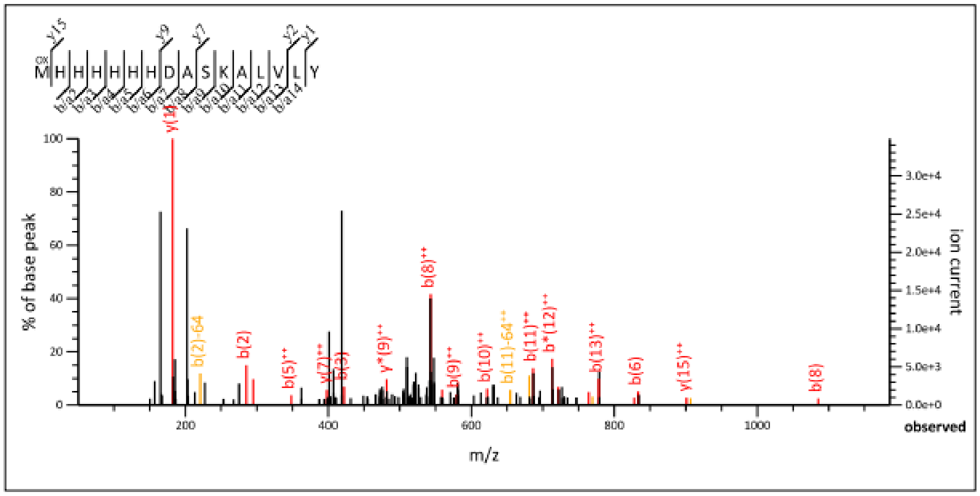

Label all possible matches ○ Label matches used for scoring ◉

```
Monoisotopic mass of neutral peptide Mr(calc): 1947.9275
Fixed modifications: Carbamidomethyl (C) (apply to specified residues or termini only)
Variable modifications:
  M1 : Oxidation (M), with neutral losses 0.0000(shown in table), 63.9983
Ions Score: 31  Expect: 0.00089
Matches : 30/210 fragment ions using 67 most intense peaks   (help)
```

#	a	a++	a*	a*++	b	b++	b*	b*++	Seq.	y	y++	y*	y*++	#
1	120.0478	60.5275			148.0427	74.5250			M					16
2	257.1067	129.0570			**285.1016**	143.0544			H	1801.8993	***901.4533***	1784.8728	892.9400	15
3	394.1656	197.5864			**422.1605**	211.5839			H	1664.8404	***832.9239***	1647.8139	824.4106	14
4	531.2245	266.1159			**559.2194**	280.1133			H	1527.7815	***764.3944***	1510.7550	755.8811	13
5	668.2834	334.6453			696.2783	***348.6428***			H	1390.7226	695.8649	1373.6961	687.3517	12
6	805.3423	403.1748			**833.3372**	417.1723			H	1253.6637	627.3355	1236.6371	618.8222	11
7	942.4012	471.7043			970.3961	485.7017			H	1116.6048	***558.8060***	1099.5782	550.2928	10
8	1057.4282	529.2177			**1085.4231**	***543.2152***			D	979.5459	490.2766	962.5193	481.7633	9
9	1128.4653	564.7363			1156.4602	***578.7337***			A	864.5189	432.7631	847.4924	424.2498	8
10	1215.4973	608.2523			1243.4922	***622.2498***			S	793.4818	***397.2445***	776.4553	388.7313	7
11	1343.5923	672.2998	1326.5657	663.7865	1371.5872	***686.2972***	1354.5606	677.7840	K	706.4498	353.7285	689.4232	345.2153	6
12	1414.6294	707.8183	1397.6028	699.3051	1442.6243	***721.8158***	1425.5978	713.3025	A	**578.3548**	289.6811			5
13	1527.7135	764.3604	1510.6869	755.8471	1555.7084	***778.3578***	1538.6818	769.8446	L	507.3177	254.1625			4
14	1626.7819	813.8946	1609.7553	805.3813	1654.7768	***827.8920***	1637.7502	819.3788	V	394.2336	197.6205			3

sPPO C-terminal Mass Spectra

Mascot Search Results

Peptide View

MS/MS Fragmentation of **AEDFAKLSYKKAL**
Found in **sPPO** in **sPPO**, sPPO

Match to Query 24664: 1482.807885 from(495.276571,3+) intensity(48174975.9063) rtinseconds(1743.867767) index(15483)
Title: 20231207_sPPO.17322.17322.3
Data file C:\Users\EKYSC\Desktop\20231207_sPPO.raw

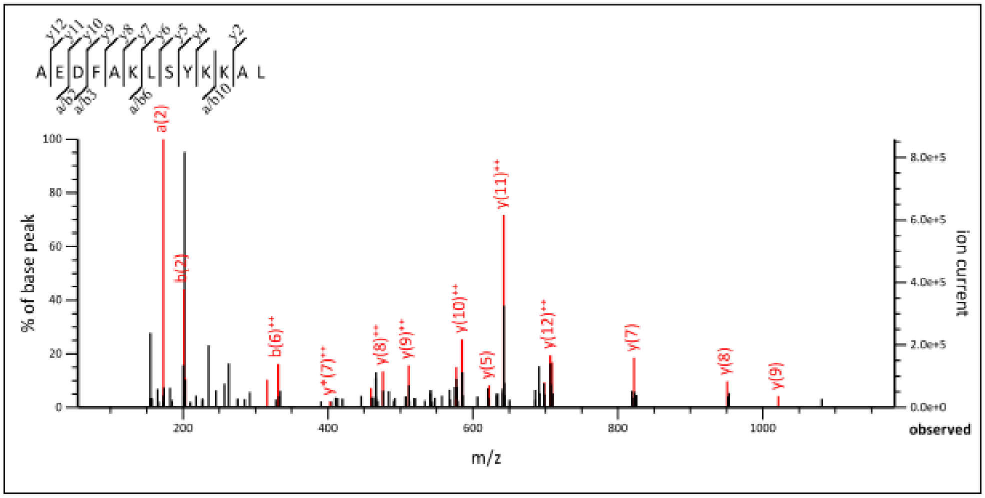

Label all possible matches ○ Label matches used for scoring ◉

Monoisotopic mass of neutral peptide Mr(calc): 1482.8082
Fixed modifications: Carbamidomethyl (C) (apply to specified residues or termini only)
Ions Score: 32 **Expect:** 0.00067
Matches : 21/120 fragment ions using 57 most intense peaks (help)

#	a	a++	a*	a*++	b	b++	b*	b*++	Seq.	y	y++	y*	y*++	#
1	44.0495	22.5284			72.0444	36.5258			A					13
2	173.0921	87.0497			201.0870	101.0471			E	1412.7784	706.8928	1395.7518	698.3796	12
3	288.1190	144.5631			316.1139	158.5606			D	1283.7358	642.3715	1266.7093	633.8583	11
4	435.1874	218.0974			463.1823	232.0948			F	1168.7089	584.8581	1151.6823	576.3448	10
5	506.2245	253.6159			534.2195	267.6134			A	1021.6404	511.3239	1004.6139	502.8106	9
6	634.3195	317.6634	617.2930	309.1501	662.3144	331.6608	645.2879	323.1476	K	950.6033	475.8053	933.5768	467.2920	8
7	747.4036	374.2054	730.3770	365.6921	775.3985	388.2029	758.3719	379.6896	L	822.5084	411.7578	805.4818	403.2445	7
8	834.4356	417.7214	817.4090	409.2082	862.4305	431.7189	845.4040	423.2056	S	709.4243	355.2158	692.3978	346.7025	6
9	997.4989	499.2531	980.4724	490.7398	1025.4938	513.2506	1008.4673	504.7373	Y	622.3923	311.6998	605.3657	303.1865	5
10	1125.5939	563.3006	1108.5673	554.7873	1153.5888	577.2980	1136.5623	568.7848	K	459.3289	230.1681	442.3024	221.6548	4
11	1253.6888	627.3481	1236.6623	618.8348	1281.6838	641.3455	1264.6572	632.8322	K	331.2340	166.1206	314.2074	157.6074	3
12	1324.7260	662.8666	1307.6994	654.3533	1352.7209	676.8641	1335.6943	668.3508	A	203.1390	102.0731			2
13									L	132.1019	66.5546			1
